# Refining Mouse Models of Gaucher Disease: Advancing Mechanistic Insights, Biomarker Discovery, and Therapeutic Strategies

**DOI:** 10.3390/ijms262411915

**Published:** 2025-12-10

**Authors:** Nima Fattahi, Jiapeng Ruan, Glenn Belinsky, Shu Xing, Pramod K. Mistry, Shiny Nair

**Affiliations:** Department of Internal Medicine, Yale School of Medicine, New Haven, CT 06510, USA; nima.fattahi@yale.edu (N.F.); jiapeng.ruan@yale.edu (J.R.); glenn.belinsky@yale.edu (G.B.); shu.xing@yale.edu (S.X.)

**Keywords:** disease, Gaucher, lysosomal storage disease, glucosylceramide, biomarker

## Abstract

Gaucher disease (GD), caused by biallelic pathogenic variants in *GBA1*, has evolved from being understood as a macrophage-restricted lysosomal disorder to a multisystem condition involving neuroinflammation, immune dysregulation, and cell-type-specific lipid toxicity. This expanded view has driven a parallel progression in GD mouse model development. Early chemically induced and germline knockout models provided foundational insights but were limited by perinatal lethality or incomplete phenotypic fidelity. Subsequent generations of conditional, inducible, and lineage-specific models enabled dissection of visceral and neuronopathic manifestations and clarified the contributions of macrophages, B cells, neurons, microglia, osteoblasts, and endothelial cells to disease pathogenesis. More recent humanized immune and gene-edited platforms, together with multi-omics integration, now allow modeling of genotype-specific biology and therapeutic response with greater translational precision. In this review, we synthesize the evolution of GD mouse models across these eras, evaluate their strengths and limitations, and highlight species-specific challenges including differences in lipid metabolism, immune architecture, and the absence of the *GBAP1* pseudogene in mice that influence interpretation and clinical translation. We outline emerging strategies for incorporating patient-derived mutations, modifier pathways, and clinically meaningful endpoints into future models. Our aim is to provide a coherent framework that bridges murine and human GD biology and supports the development of more predictive platforms to accelerate mechanistic discovery, biomarker development, and therapeutic innovation across all subtypes of GD.

## 1. Introduction

Gaucher disease (GD) is an autosomal recessive lysosomal storage disorder caused by biallelic mutations in *GBA1*, the gene that encodes the lysosomal enzyme β-glucocerebrosidase (GCase, EC 3.2.1.45). Reduced GCase activity results in accumulation of its lipid substrates, glucosylceramide (GlcCer) and glucosylsphingosine (GlcSph) within the lysosomes, most conspicuously in cells of the mononuclear phagocyte system. Clinically, GD has long been classified into three subtypes based on the presence and age of onset of neurologic involvement. Type 1 (GD1) is the chronic, non-neuronopathic form and is dominated by visceral and hematologic disease (hepatosplenomegaly, cytopenias, bone disease); GD1 patients generally respond well to systemic treatments such as enzyme replacement therapy (ERT); prior to the advent of ERT hematopoietic stem cell transplantation (HSCT) was used in selected patients. Type 2 (GD2) is an acute neuronopathic infantile form with rapid neurodegeneration and early mortality, while Type 3 (GD3) is a more slowly progressive, chronic neuronopathic form with both visceral and neurologic manifestations emerging in childhood or adolescence or even in adulthood [1]. These categories are useful clinically but do not capture the full biological continuum of *GBA1*-related phenotypes. GD1 patients have increased risk of Parkinson’s disease. Long before large registry-based analyses were possible, the Yale prospective cohort [2] provided the first rigorous estimate of Parkinson’s disease risk in Gaucher disease. In 444 consecutively evaluated patients with GD1 followed over 12 years, the lifetime relative risk of PD was 21.4-fold higher than expected from population norms, with a trend toward male predominance [2]. This study established definitively that PD is not a rare coincidence in GD but an authentic phenotypic extension of the disease. Our new ICGG Registry analysis expands on these foundational observations using a global cohort of 1618 patients with GD1 and longitudinal neurologic data captured through standardized case report forms By age 80, one in nine patients had clinically confirmed PD, and more than one in five exhibited PD, DLB, or at least two significant parkinsonian features, which we defined as possible parkinsonian syndrome (pPS) (Alcalay R et al., in review). Given that registry-based assessments often underestimate neurological outcomes, these figures likely represent lower-bound penetrance estimates. Together, the Yale cohort and the ICGG dataset show consistent, convergent evidence: GD1 confers a substantial elevation in PD risk, far above background, but penetrance does not appear to scale simply with degree of GCase deficiency. This discordance indicates that *GBA1*-linked PD is strongly shaped by age-related attrition of GCase activity and modifiers that remain to be elucidated.

Historically, GD was framed as a predominantly macrophage-centric disease because Gaucher cells (lipid-laden macrophages) are a prominent histologic hallmark and because macrophage-targeted therapies substantially ameliorate systemic features. Yet work over the past two decades has broadened this view: GD is a multisystem disorder that affects osteoblasts, endothelial cells, hepatocytes, B cells, neurons, and glia, and is accompanied by systemic immune dysregulation, hepatic and skeletal pathology, and hematologic abnormalities [2]. In neuronopathic GD (nGD; types 2 and 3), CNS pathology such as microglial activation, astrogliosis, neuronal dysfunction and loss, and neuroinflammation plays a central role and cannot be addressed by interventions directed at the hematopoietic system because brain microglia originate from yolk-sac progenitors and are not replaced by HSCT [3,4].

Mouse models have been indispensable for dissecting GD biology and for preclinical testing of therapies, but the diversity of human GD phenotypes requires an equally diverse set of animal models. Early strategies included chemically induced substrate accumulation and germline *Gba1* knockouts; many of these provided important mechanistic insights but were limited by perinatal lethality or incomplete recapitulation of human pathology [5]. To model visceral GD1, investigators developed conditional and chimeric approaches that preserve survival while producing progressive macrophage-rich visceral disease (hepatosplenomegaly, cytopenias, bone involvement) suitable for testing systemic therapies [6]. Examples include myeloid-targeted deletions, genetic chimeras, and rescue strategies that selectively restore GCase activity in the CNS to unmask peripheral disease [7]. These “visceral” models aim to mirror the chronic, treatment-responsive systemic phenotype of GD1 and to provide platforms for evaluating ERT, substrate-reduction therapies, and systemic gene therapies.

By contrast, modeling neuronopathic GD requires CNS-directed approaches. Conditional knockouts that delete *Gba1* in neural lineages (e.g., Nestin-Cre) or in specific cell types (neurons vs. microglia), inducible systems, and combinatorial genetic backgrounds have clarified cell-type contributions to nGD [8]. Recent cell-specific studies show that neuronal GCase loss can precipitate rapid neurodegeneration and seizures, whereas microglial GCase deficiency promotes substrate accumulation and a slower, inflammatory phenotype demonstrating that neurons and microglia play distinct but interacting roles in nGD pathogenesis [4]. Viral-vector strategies and humanized knock-in alleles further permit modeling of mutation-specific biology and of therapeutic gene delivery to the CNS.

Despite these advances, translational limitations persist. Mice lack key human genomic features, most notably the *GBAP1* pseudogene which influences recombination landscapes, transcript complexity, and pathogenic variant formation. Species-specific differences in lipid metabolism, immune architecture, and lifespan further constrain model fidelity and therapeutic prediction. Accordingly, no single model captures the full spectrum of human GD; rather, visceral, neuronopathic, inducible, cell-specific, and humanized complementary models are required to address distinct mechanistic and translational questions.

### Scope and Purpose of This Review

In this review, we (i) provide a historical overview of GD mouse model development, from chemical and germline strategies to modern conditional, inducible, and humanized systems; (ii) categorize and critically assess models based on design, targeted tissues, and relevance to visceral versus neuronopathic phenotypes; (iii) synthesize insights into cell-type–specific contributions of macrophages, B cells, hepatocytes, neurons, microglia, and other lineages; (iv) discuss species-specific limitations that shape interpretation and translational applicability; (v) highlight contemporary advances including humanized models and multi-omics integration; and (vi) propose best practices and future directions for refining model fidelity and predictive value. Our aim is to provide a coherent, critically informed framework to guide investigators in selecting, refining, and interpreting GD mouse models across the full clinical and biological spectrum of the disease.

## 2. Genomic Landscape of the *GBA1* Locus in Humans and Mice

**Figure 1:**
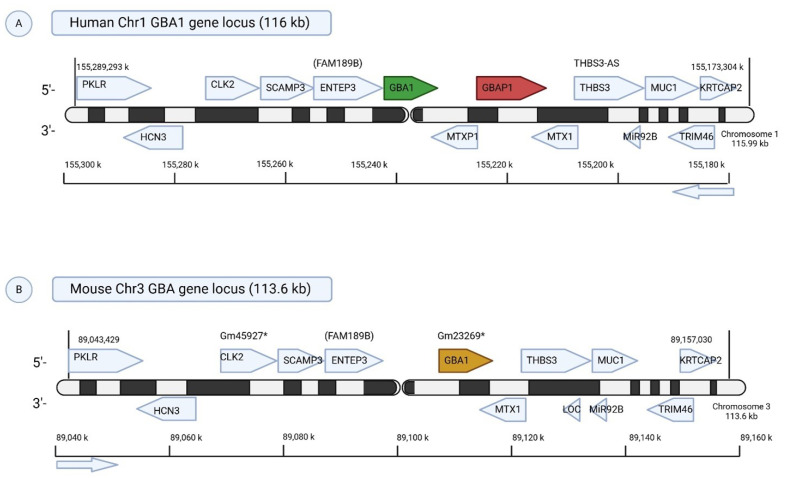
**Comparative Genomic Organization of the *GBA1* Locus in Humans and Mice.** (**A**) Human Chr1q21 (116 kb): The human *GBA1* gene is located on chromosome 1q21 and is flanked by several protein-coding genes involved in glycolysis (PKLR), cell cycle and splicing (CLK2), endosomal trafficking (SCAMP3, ENTEP3), mitochondrial import (MTX1, MTXP1), and neuronal function (TRIM46). A notable feature is the nearby *GBAP1* pseudogene, which shares high homology with *GBA1* and can contribute to pathogenic mutations via gene conversion. (**B**) Mouse Chr3 (113.6 kb): The murine *Gba1* locus, although syntenic to the human locus, lacks *GBAP1*, representing a key structural difference. Most flanking genes are conserved, though mice carry predicted genes (e.g., Gm45927, Gm23269) with unknown functions. These differences may impact gene regulation and disease modeling in Gaucher disease research. * represents predicted mouse gene with no well-defined function yet.

The *GBA1* gene encodes the lysosomal enzyme β-glucocerebrosidase (GCase) and its deficiency underlies Gaucher disease. In humans, *GBA1* is located on chromosome 1q21, within one of the most gene-dense and structurally complex regions of the human genome. The locus spans approximately 133 kilobases and is flanked by 15 genes, including the highly homologous pseudogene *GBAP1* [9]. This pseudogene shares over 96% sequence identity with *GBA1*, particularly in the coding region, and poses significant challenges for molecular diagnostics due to frequent recombination events. Gene conversion between *GBA1* and *GBAP1* can result in pathogenic mutations such as the p. Leu483Pro (legacy nomenclature L444P) mutation being transferred into the functional gene, leading to severe forms of GD [10] (Figure 1).

In contrast, the mouse *Gba1* gene resides on murine chromosome 3 and notably lacks a *GBA* pseudogene [11], creating key differences in genomic architecture that impact modeling of human mutations and recombination dynamics. Adjacent to *Gba1* in the mouse is *Mtx1* (Metaxin 1), a ubiquitously expressed gene essential for mitochondrial protein import and embryonic development [12]. This proximity imposes additional constraints on genetic targeting strategies, particularly when engineering conditional or knock-in alleles (Figure 1).

Despite structural similarities, with both human and murine *GBA1* loci containing 10 exons and high homology, the phenotypic expression of GD is highly variable, and it is influenced by complex genetic and epigenetic modifiers. Residual GCase activity, the presence of modifier genes, and interactions with broader metabolic and inflammatory pathways contribute to the heterogeneous clinical spectrum [13]. For instance, the p.Asn409Ser (legacy nomenclature, N370S) mutation is commonly associated with type 1 GD, while homozygosity for p.Leu483Pro (L444P) is linked to neuronopathic subtypes (types 2 and 3). The p.Asp447His (D409H) mutation has been specifically implicated in type 3c GD, which additionally features cardiovascular calcifications, including aortic and valvular involvement [14].

Historically, research efforts focused on macrophage pathology, contributing to the development of enzyme replacement therapy (ERT) and the identification of macrophage-derived biomarkers such as chitotriosidase, CCL18, and gpNMB. However, with the advent of more refined tools such as in situ enzyme activity probes, lysosome enrichment techniques, and multi-omics platforms, our understanding of genotype-phenotype relationships and cell-type-specific pathology is becoming increasingly nuanced [15,16].

The development of genetically engineered mouse models (GEMMs) in the 1980s, through embryonic stem cell manipulation, revolutionized the study of lysosomal storage disorders [17]. These models enabled precise in vivo studies of gene function and facilitated the investigation of disease mechanisms and therapeutic interventions. Although mice offer many advantages including physiological tractability, defined genetics, and short reproductive cycles, they also exhibit important species-specific differences. These include higher metabolic rates, shorter lifespans, strain-specific immune responses [18], and the absence of *GBAP1*, which together limit the extrapolation of some findings to human disease.

Nevertheless, mouse models have been instrumental in the preclinical development of nearly all current and emerging GD therapies. ERT has demonstrated efficacy in type 1 GD [1], and studies using neuronopathic GD (nGD mice have shown improved survival with intracerebroventricular delivery of Imiglucerase [19]. Substrate reduction therapy (SRT) was also pioneered in GD mouse models, informing the clinical introduction of Eliglustat for type 1 GD and ongoing trials of brain-penetrant SRT agents for nGD [20,21,22,23]. Additional therapeutic strategies, including bone marrow transplantation, gene therapy [6], and chemical chaperones [24]. Transplantation of genetically modified hematopoietic stem cells has shown promise in mouse models and is under active investigation in human trials.

By recapitulating key features of GD pathophysiology and therapeutic response, mouse models remain indispensable tools for mechanistic discovery and translational research. In this review, we chart the evolution of GD mouse models through four distinct eras of development, highlighting our experimental contributions and outlining strategies to enhance their translational impact in future research.

## 3. Early Models: Chemically Induced Approaches

The earliest experimental model of GD was established in 1975 by Kanfer and colleagues, who demonstrated that conduritol-β-epoxide (CBE), an irreversible inhibitor of β-glucosidase, could recapitulate hallmark features of GD in mice [25]. Daily administration of CBE at 100 mg/kg to adult BALB/c male mice led to a greater than 90% reduction in GCase activity, resulting in the pathological accumulation of GlcCer in multiple organs, including the spleen, liver, and brain. Notably, the brain exhibited the highest sensitivity to substrate buildup, making the model particularly useful for investigating both visceral and nGD pathophysiology (Table 1).

This broad, multisystem involvement allowed researchers to explore therapeutic strategies such as adenoviral-mediated gene therapy to restore GCase activity and reduce GlcCer accumulation [26]. Subsequent studies using CBE models revealed critical insights into nGD mechanisms. For example, they uncovered the role of receptor-interacting protein kinase 3 (RIPK3)-mediated necroptosis in neurodegeneration and demonstrated that genetic background significantly influences disease progression [27]. In one study, 25 mg/kg CBE administered to 15 different inbred mouse strains resulted in survival times ranging from 50 to 200 days, highlighting the potential contribution of genetic modifiers to GD severity [28].

However, the interpretation of strain-dependent outcomes in chemically induced models is complicated by pharmacokinetic (PK) and pharmacodynamic (PD) variability across mouse strains. Differences in drug absorption, metabolism, distribution, and clearance can affect CBE exposure and efficacy, potentially confounding the attribution of phenotypic variability to genetic susceptibility alone [28]. These factors are significant confounders in chemical models compared to genetic models because the observed phenotype is a product of both the drug’s effect and the animal’s biology. In genetic models, the disease-causing mutation is a stable, intrinsic factor. In chemical models, strain-specific differences in enzymes or transporters that handle CBE can lead to vastly different effective drug concentrations at the target site, creating phenotypes that mimic more severe or conversely milder phenotypic expression. Without controlling for putative PK and PD parameters, it remains unclear whether observed differences reflect intrinsic genetic modifiers of GD or extrinsic variations in drug handling.

Despite these challenges, CBE-based models have been instrumental in biomarker discovery. Beyond pathophysiology, CBE models have been instrumental for biomarker discovery. Glycoprotein non-metastatic B (GPNMB), elevated in both GD spleen and microglia, emerged as a robust cross-species biomarker [36,37]. In CBE-treated mice (100 mg/kg for 28 days), GPNMB levels were significantly increased in the brain and correlated with glial activation and disease severity [38]. Unlike humans, mice do not express key macrophage biomarkers such as chitotriosidase or CCL18, positioning GPNMB as a valuable translational indicator for murine GD and related lysosomal disorders.

A well-recognized limitation of the CBE model is that it does not activate the unfolded protein response (UPR), as pharmacological inhibition of GCase allows the protein to traffic normally to lysosomes without inducing misfolding [39]. This distinguishes CBE-induced pathology from knock-in genetic models of GD [33], where mutant GCase often accumulates in the endoplasmic reticulum and triggers UPR. Nevertheless, CBE models provide a reliable and tunable platform to study substrate accumulation, neuroinflammation, and the effects of timed therapeutic interventions. For example, high-dose CBE treatment initiated at postnatal day 8 induces acute neuronopathic features, while prolonged low-dose regimens (<25 mg/kg) have been used to model chronic GD and its pathological overlap with Parkinson’s disease (PD) [27].

In summary, chemically induced GD models, particularly those using CBE, have been foundational in elucidating disease mechanisms, identifying biomarkers, and evaluating therapies. Their reproducibility, multisystem involvement, and capacity to model neurodegeneration make them indispensable tools in GD research. However, careful consideration of genetic background and PK/PD parameters is essential to maximize their translational relevance and interpret findings within a biologically meaningful framework (Table 1).

## 4. *Gba1* Conditional Knockout Mouse Models in Hematopoietic Cells (*Gba1*^fl/fl^; Mx1-Cre^+^)

To model GD in adult mice while avoiding embryonic lethality, Karlsson’s laboratory developed a conditional knockout system by crossing *Gba1*^fl/fl^ mice with Mx1-Cre transgenics. GCase deletion was induced postnatally using polyinosinic-polycytidylic acid (poly I:C), which activates the Mx1 promoter primarily in hematopoietic cells, leading to the conditional deletion of exons 9–11 of the *Gba1* gene [6]. This approach enabled progressive disease modeling with preserved embryonic GCase expression. These *Gba1*^fl/fl^; Mx1-Cre mice exhibited hallmark features of hematologic and visceral GD, including Gaucher cell infiltration, splenomegaly, and cytopenia. GCase deficiency was most pronounced in the bone marrow, spleen, and liver, while the brain remained largely unaffected. By 12–16 months post-induction, mice developed severe splenomegaly (up to 10-fold), GlcCer accumulation, microcytic anemia, and splenic infarctions paralleling human GD pathology (Table 1).

Building on this model, our group enhanced the conditional strategy by targeting *Gba1* exons 8–11, achieving >95% GCase depletion in hematopoietic and mesenchymal stem cells [2]. These mice exhibited severe osteopenia due to impaired osteoblastogenesis driven by elevated GlcSph but not increased bone resorption. This bone defect was corroborated in GD patient-derived iPSCs [40] and aligns with clinical observations of low peak bone mass in children with GD, a modifiable risk factor for adult osteopenia and responsive to ERT [41]. Unexpectedly, GCase deficiency also disrupted T-cell and dendritic cell development, revealing roles beyond macrophage dysfunction [42]. These GD mice exhibited other features including growth retardation, gibbus deformity (a hallmark of GD type 3), and elevated GlcCer and GlcSph levels in visceral tissues. Although mice lacked neurological symptoms, they recapitulated key metabolic abnormalities observed in human GD, including reduced HDL, and elevated liver enzymes. Despite significant macrophage activation, chitotriosidase was not upregulated in GD mouse models, reflecting the low or absent expression of *Chit1* in murine macrophages a key species-specific difference that complicates biomarker translation from human to mouse systems. Instead, glycoprotein non-metastatic melanoma protein B (gpNMB) emerged as a robust biomarker through transcriptomic profiling and was subsequently validated in human GD cohorts. Establishing gpNMB as GD biomarker reflects the evolution of GD research: from relying on known human biomarkers to discovering murine-specific correlates and then translating them back to human studies [2,37]. Histologically, these mice exhibited extensive storage cell accumulation in the liver, spleen, bone marrow, lymph nodes, and thymus, with evidence of extramedullary hematopoiesis contributing to organomegaly. Indeed extramedullary hematopoiesis has also been described in human GD [43,44]. The *Gba1*^fl/fl^ Mx1-Cre^+^ mouse model has been pivotal in recapitulating hematologic and visceral features of GD, uncovering GCase’s role in osteoblast function, immune cell development, and skeletal pathology. It has enabled biomarker discovery (e.g., gpNMB, GlcSph) and served as a valuable platform for testing therapeutic approaches, including gene therapy [36,37].

One of the most important translational insights gained from hematopoietic-specific Gaucher disease models has been the elucidation of the link between chronic lysosomal lipid accumulation and B-cell neoplasia. Clinical studies have consistently shown an elevated incidence of monoclonal gammopathy of undetermined significance (MGUS) and multiple myeloma in patients with GD [45,46,47]. Conditional hematopoietic models have proven instrumental in probing this association, allowing for detailed investigation of B-cell pathology in the context of myeloid storage disease. Using related murine models, Pavlova et al. reported B-cell lymphoma and myeloma in Gaucher mice, linking chronic myeloid storage disease to B-cell neoplasia [48]. Mechanistic studies showed that pathological sphingolipids in GD are recognized by type II natural killer T (NKT) cells, which provide aberrant help to B cells and drive production of lipid-specific antibodies [49]. This antigen-driven pathway establishes a direct link between lysolipid accumulation, chronic B-cell activation, and the markedly elevated (~30-fold) risk of gammopathy and myeloma in GD [50]. Together, these findings define a pathogenic axis in which persistent lysosomal lipid storage fuels immune dysregulation, creating a chronic inflammatory microenvironment that fosters clonal B-cell expansion and malignant progression. These insights underscore the potential for antigen-specific or immune-modulatory strategies to prevent hematologic malignancies in this high-risk population (Table 1).

## 5. Modeling Hematopoietic and Immune Dysregulation in Gaucher Disease Using the Vav-Cre *Gba1*^fl/fl^ Mouse

The Vav-Cre *Gba1*^fl/fl^ model was developed to provide a genetically precise and chronically viable platform for studying hematopoietic-intrinsic immune dysregulation in Gaucher disease [33]. Unlike the Mx1-Cre system which induces *Gba1* deletion postnatally via poly(I:C), Vav-Cre drives recombination during early embryogenesis in hematopoietic progenitors, thereby modeling the developmental onset of GCase deficiency in a manner that is more physiologically aligned with human disease. This early and lineage-specific deletion avoids the acute toxicity and systemic inflammation associated with interferon-driven Cre induction and allows for long-term interrogation of immune pathogenesis. Notably, Vav-Cre *Gba1*^fl/fl^ mice recapitulate hallmark features of GD1, including splenomegaly, cytopenias, bone marrow infiltration by Gaucher-like macrophages, and storage of GluCer and GluSph in immune tissues. Importantly, the model demonstrates profound immune dysregulation, with macrophage lysosomal dysfunction, enhanced expression of GPNMB, and altered cytokine profiles. Additional features include activated NK cells, effector CD8^+^ T cell skewing, and increased complement activation immune signatures that closely mirror those observed in human GD and underscore the contribution of hematopoietic cells to disease pathology [51]. In contrast, the Mx1-Cre *Gba1*^fl/fl^ model, which is widely used for systemic *Gba1* deletion via poly(I:C)-induced Cre recombination, produces acute, severe disease affecting both hematopoietic and non-hematopoietic compartments, such as the liver. While valuable for modeling end-stage organ pathology and rapid substrate accumulation, the Mx1-Cre system lacks cell-type specificity, is complicated by interferon-induced toxicity, and cannot distinguish the independent roles of immune versus non-immune cells in disease progression. Furthermore, its aggressive disease course limits its utility for studying chronic or progressive immune alterations. In sum, the Vav-Cre model offers a developmentally relevant and immunologically focused tool for dissecting the contributions of hematopoietic cells to GD pathogenesis. It complements global and neuronopathic models by enabling detailed exploration of immune cell phenotypes, modifier gene interactions, and sustained therapeutic effects in a setting that more closely reflects chronic human GD (Table 2).

## 6. Genetic Models of Type 2 Gaucher Disease: From Germline Knockouts to Conditional and Therapeutic Platforms

The first genetically engineered mouse model of GD, developed in 1992 by Dr. Edward Ginns and colleagues, involved the insertion of a neomycin (Neo) cassette into exons 9 and 10 of the *Gba1* gene, resulting in complete loss of GCase activity [5]. Homozygous (*Gba1^neo/neo^*) mice exhibited marked GlcCer accumulation in the liver, spleen, lungs, and brain, closely mimicking type 2 GD (GD2). However, these mice died within 24 h of birth due to severe skin barrier defects caused by impaired conversion of GlcCer to ceramide, critical for epidermal lamellae formation. This phenotype paralleled the "collodion baby" presentation in GD2 patients and highlighted the role of *Gba1* in systemic and epidermal lipid metabolism. Additional features such as poor feeding, respiratory distress, and limited movement indicated CNS involvement, though neonatal lethality restricted longer-term studies [29,30]. To overcome this limitation, the *K14-Cre-Gba1^lnl/lnl^* model was developed. By rescuing *Gba1* expression in the epidermis via *K14*-driven Cre recombinase, this model enabled postnatal survival while preserving systemic GCase deficiency. Mice survived ~10–14 days, during which they developed hallmark GD2 features, including visceral lipid accumulation, Gaucher cells, and progressive neurodegeneration. Neurological symptoms included seizures, paralysis, and abnormal gait. Histopathology revealed lipid-laden neurons, apoptotic markers, and intense neuroinflammation (GFAP^+^, Iba1^+^), faithfully modeling acute GD2 neuropathology [7,31]. Despite its utility, the model has limitations. Its rapid disease course precludes study of chronic neurodegeneration, and ~15% of mice exhibit leaky recombination in non-epidermal tissues, potentially confounding therapeutic studies. Systematic validation using qPCR, Western blot, and GCase activity assays is essential to confirm tissue-specific gene deletion and avoid misinterpreting residual enzyme activity as therapeutic benefit. The *K14-Cre-Gba1^lnl/lnl^* model has been instrumental in gene therapy development, enabling studies with AAV vectors and hematopoietic stem cell transplantation [32]. In recent work, our group leveraged the K14-Cre-*Gba1^lnl/lnl^* platform for longitudinal spatial transcriptomics and lipidomics. We identified a disease trajectory characterized by a transition from homeostatic to damage-associated microglia (DAM), reactive astrogliosis, and early blood–brain barrier (BBB) disruption. Spatial lipidomics uncovered elevated lysophosphatidylcholine (LPC), suggesting a novel mediator of neurodegeneration. Key biomarkers ApoE (lipid redistribution), NfL (neuronal injury), and GPNMB (macrophage activation) were identified as candidate endpoints for translational monitoring [4]. In summary, genetic models of GD2 from the original germline knockout to the conditional *K14-Cre-Gba1^lnl/lnl^* system have been essential for uncovering mechanisms of lipid accumulation, neuroinflammation, and therapeutic response. While limitations remain, these models continue to guide translational strategies, with refinements such as inducible knockouts and humanized alleles poised to expand their utility in modeling the full clinical spectrum of nGD (Table 1). While powerful, the K14 model has notable limitations: ~15% of mice exhibit leaky recombination in non-epidermal tissues, necessitating rigorous validation of tissue-specific *Gba1* deletion by qPCR, Western blot, and GCase assays. Additionally, the rapid disease course precludes studies of chronic or progressive pathology.

## 7. Point Knock-in Mutation Gaucher Disease Mouse Models

Point knock-in (KI) mouse models of GD have been instrumental in elucidating the mutation-specific effects of *Gba1* variants on GCase function, lipid accumulation, and disease progression. These models introduce precise, disease-associated mutations directly into the murine *Gba1* gene, enabling the in vivo recapitulation of human genotype-phenotype correlations [34]. One of the earliest approaches utilized the Single Insertion Mutagenesis Procedure (SIMP) to generate RecNciI (type 2 GD) and L444P (type 3 GD) mutations in embryonic stem (ES) cells [35]. SIMP maintains one functional allele while introducing a non-functional truncated allele, allowing controlled assessment of mutation severity. Homozygous RecNciI and L444P mice demonstrated postnatal lethality, with RecNciI mutants displaying severe phenotypes including small body size, poor skin turgor, wrinkled red skin, and absence of feeding, while L444P homozygotes exhibited milder symptoms and evidence of feeding [35]. Biochemical analysis showed GCase activity was mutation-dependent: L444P mutants retained ~20% enzymatic activity, while RecNciI mutants retained only 4–9%, correlating with more severe GlcCer accumulation and ceramide reduction, especially in the skin. To broaden the spectrum of genotype modeling, additional pathogenic *Gba1* missense mutations p.Asn370Ser (N370S), p.Val394Leu (V394L), p.Asp409His (D409H), and p.Asp409Val (D409V) were introduced via targeted homologous recombination [34]. While N370S^+neo^ homozygotes lacked detectable GCase mRNA or enzymatic activity and succumbed to severe epidermal barrier dysfunction within 24 h of birth, V394L, D409H, and D409V homozygotes exhibited near-normal gross behavior and survived up to 58–78 weeks. Phenotypic severity increased in point-mutant/null combinations, demonstrating a dose-dependent relationship between residual GCase activity and storage pathology. Histological analyses revealed Gaucher cell infiltration in the spleen, liver, and lungs, particularly prominent in D409V^null^ mice, with Mac-3 immunostaining confirming macrophage involvement. accumulation was detected in visceral tissues, with D409V/null mice showing the most pronounced lipid storage, followed by D409H/null and V394L/null variants. These point KI models have provided valuable insights into how specific *GBA1* mutations influence GD progression [33]. While point knock-in (KI) models are designed for precision, they often reveal profound species-specific differences that limit their ability to recapitulate human genotype-phenotype correlations. A striking example is the unexpected lethality of homozygous p.Asn409Ser (legacy N370S) mice, which die within 24 h of birth due to severe skin permeability defects. This contrasts sharply with humans, in whom p.Asn409Ser is the most common mutation associated with type 1 GD, a non-neuronopathic form, typically mild form of the disease compatible with a near-normal lifespan. This species-specific discrepancy likely reflects fundamental differences in murine versus human skin physiology and lipid homeostasis, complicating efforts to model late-onset or chronic GD phenotypes. Similarly, p.Asp448His (legacy D409H) homozygous mice, which develop visceral Gaucher cell infiltration, do not display the hallmark cardiac calcifications seen in human GD type 3c (GD3c), despite this mutation being strongly linked to valvular and vascular pathology in patients [33]. These examples highlight the broader challenge of faithfully recapitulating human genotype-phenotype relationships in murine models. Differences in skin architecture, cardiac biology, and lipid handling can drastically reshape phenotypic outcomes. 

To overcome these barriers, future studies may benefit from:

(1) humanized mouse models carrying the full human *GBA1* and *GBAP1* loci under native regulatory elements;

(2) iPSC-derived organotypic systems (e.g., cardiomyocytes or keratinocytes) for cell-type specific modeling; and

(3) modifier gene discovery platforms to elucidate pathways responsible for species-specific phenotypic divergence.

Recognizing these limitations is essential to guide refinement of GD mouse models and improve the translational relevance of therapeutic investigations (Table 1). 

## 8. Dissecting Cell-Type-Specific Roles in Neuronopathic GD: Neurons as Primary Drivers and Microglia as Modulators

A central question in nGD pathogenesis is whether pathology is driven by cell-autonomous dysfunction within specific CNS lineages or by non-autonomous, paracrine effects between them. The development of conditional knockout and, more powerfully, conditional rescue models has enabled a direct dissection of the roles of neurons and glia, moving beyond correlation to establish causality. 

## 9. *Gba1* Conditional Knockout in Neuronal and Macroglial Cells (*Gba1*^fl/fl^ Nestin-Cre)

Early attempts to parse these contributions utilized Cre-loxp systems to delete in specific lineages. The *Gba1*^fl/fl^ Nestin-Cre mouse model was developed to selectively disrupt GCase activity in neurons and macroglia including astrocytes, oligodendrocytes, and ependymal cells while preserving GCase function in microglia. Nestin-Cre drives recombination in neural progenitor cells (NPCs) during development, which give rise to both neurons and macroglia, but not to yolk-sac-derived microglia. This design enables the study of neuron- and macroglia-specific GCase deficiency without altering microglial function. Compared to K14-Cre; *Gba1*^fl/fl^ mice, which lack GCase in all CNS cell types and show early, severe neurodegeneration, *Gba1*^fl/fl^ Nestin-Cre mice exhibit a delayed onset of neurological symptoms (~P14–21) and slower disease progression, with paralysis developing over ~7–10 days and end-stage reached around 3 weeks of age. In contrast, K14-Cre mice become symptomatic by ~P10, progress to paralysis within 3–4 days, and die by ~2 weeks of age. Histopathological analysis reveals neuronal loss, astrogliosis, and microgliosis in both models, but lipid-engorged microglia and higher GlcCer accumulation are only observed in K14-Cre mice, supporting a protective role for microglial GCase activity [7]. These findings highlight the neuroprotective function of microglia in modulating disease severity. While neuronal and macroglial GCase loss is sufficient to cause neurodegeneration, additional loss in microglia accelerates pathology. Notably, Nestin-Cre models carry a known risk of germline recombination, potentially converting conditional alleles into global knockouts; thus, rigorous validation of recombination patterns and breeding strategies is essential to ensure cell-type specificity (Table 1).

## 10. *Gba*1 Conditional Rescue Mouse Models in Microglia and Neurons (nGDCx3cr1^Cre/+^ and nGDNes^Cre/+^)

To elucidate the distinct roles of microglia and neurons in the pathogenesis of nGD, two innovative cell-type–specific rescue models were developed: nGDCx3cr1^Cre/+^ mice, with *Gba1* restoration restricted to microglia, and nGDNes^Cre/+^ mice, with *Gba1* restoration restricted to neurons [4]. These models were derived by crossing *Gba1*^fl/fl^ mice with either Cx3cr1-Cre or Nestin-Cre drivers and represent powerful tools to interrogate the cell-autonomous and non-autonomous mechanisms of neurodegeneration in nGD. Restoration of *Gba1* in microglia led to a marked, >2-fold extension of survival, partial restoration of homeostatic microglial identity, and reduced infiltration of CCR2^+^ monocyte-derived macrophages, and peripheral immune cells. However, neuroinflammatory signatures, including IL-1β accumulation in microglia and downstream activation of astrocytes and natural killer (NK) cells, persisted, especially at later stages, suggesting that microglial *Gba1* alone delays but does not halt neurodegeneration [4]. In contrast, neuronal *Gba1* restoration dramatically prolonged survival (~200 days), fully abrogated microglial activation and immune cell infiltration, and normalized lipid storage, as evidenced by bulk and spatial lipidomics. Single-nucleus RNA-seq revealed that neuronal *Gba1* rescue corrected lysosomal, interferon-stimulated genes (ISGs), chemokine, and ApoE-driven transcriptional dysregulation across diverse brain cell types, including astrocytes, oligodendrocytes, and Purkinje neurons. Despite persistent downregulation of homeostatic microglial markers, nGD Nes^Cre/+^ brains lacked the hallmarks of neuroinflammation seen in nGD or nGDCx3cr1^Cre/+^ models, including ROS accumulation and lipid-laden astrocytes and microglia. Importantly, even in the setting of intact microglial *Gba1*, as in 6-week-old nGDCx3cr1^Cre/+^ mice, continued neuronal *Gba1* deficiency led to reactivation of disease-associated microglia (DAM and inflammatory transcriptomes, indicating that neuronal pathology can drive non-cell-autonomous glial responses over time [4]. Together, these models provide compelling evidence that neuronal *Gba1* deficiency is the primary driver of nGD-associated neuroinflammation and neurodegeneration, while microglial *Gba1* contributes to mitigating early pathology. They provide a tractable framework for understanding the temporal and cellular origins of neurodegenerative cascades in lysosomal storage disorders, as well as for designing precise therapeutic strategies that target neuron-glia interactions (Table 1). 

## 11. Cell-Type Specific Roles of GCase in Neurodegeneration: Insights from Microglial and Neuronal Deletion Models

To dissect the cell-intrinsic role of GCase deficiency in microglia, we developed the *Gba ^loxp/loxp^; Cx3cr1^Cre/+^* mouse model, enabling selective deletion of *Gba1* in microglia while sparing other neural cell types. This model was designed to determine whether microglial GCase loss alone can drive the neuroinflammatory and neurodegenerative features observed in nGD, independent of neuronal or systemic contributions. Although young mice appeared clinically unaffected, they exhibited marked accumulation of GluCer and GlcSph in the brain and serum, indicating that GCase deficiency in microglia leads to substrate buildup without immediate behavioral symptoms. With age (~12–14 months), these mice developed clear signs of neurodegeneration, including motor coordination deficits on beam-walk testing and increased turnover of microglia, coupled with recruitment of peripheral CCR2⁺ monocytes, hallmarks of neuroinflammation [4]. Importantly, aged *Gba ^loxp/loxp^; Cx3cr1^Cre/+^* mice exhibited significantly elevated serum neurofilament light chain (NfL), a sensitive and clinically validated biomarker of axonal injury and neurodegeneration. This increase in NfL paralleled that observed in adult non-neuronopathic GD (GD1) patients, further supporting the translational relevance of the model. Additional evidence for neurodegeneration came from scRNA-seq of FACS-sorted brain microglia, which showed a loss of homeostatic signatures and emergence of pro-inflammatory DAM, including upregulation of *Apoe*, *Spp1*, *Ccl3*, *Tnf*, and *Il1b*. Transcriptomic analyses also revealed a unique *Gba*-associated microglial cluster (expressing *Mtx1*, *Thbs3*) and enrichment of chemokines involved in leukocyte recruitment, suggesting a mechanistic link between microglial GCase loss and chronic neuroimmune activation. Therapeutic intervention using a brain-penetrant glucosylceramide synthase (GCS) inhibitor, GZ-161, reduced GluCer/GlcSph levels, reversed microglial DAM signatures, and suppressed NK cell activation, functionally linking substrate accumulation to progressive neuroimmune dysfunction [4].

**Figure 2:**
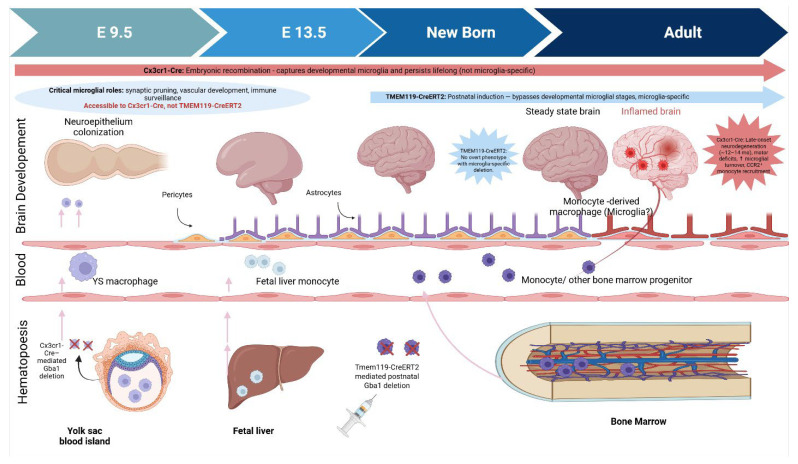
**Ontogeny of Brain-Resident Macrophages and Temporal Impact of Cre-loxP Strategies for Microglial *Gba1* Deletion.** These schematic contrasts two Cre-loxP models used to delete Gba1 in microglia. Cx3cr1-Cre drives embryonic recombination (E9.5), capturing early microglial progenitors but also other myeloid cells. This model reveals late-onset neurodegeneration with microglial turnover and monocyte infiltration. Tmem119-CreERT2, induced postnatally, achieves microglia-specific recombination but bypasses developmental stages; mice show no overt phenotype. The developmental window shaded (E9.5–birth) highlights critical microglial functions—accessible to Cx3cr1-Cre but not Tmem119-CreERT2underscoring how timing and specificity shape disease modeling outcomes.

Supporting the broader conclusion that neurons are the principal drivers of Gaucher-associated neurodegeneration, a recent study by Duffy et al. employed tamoxifen-inducible, cell-type–specific *Gba1* knockouts and demonstrated that postnatal neuron-specific deletion alone was sufficient to cause weight loss, motor dysfunction, early lethality, and widespread neurodegeneration [52]. In contrast, microglia-specific *Gba1* deletion (using TMEM119^CreERT2^) produced no overt phenotype. Their findings reinforce the neuronal GCase in maintaining CNS integrity and suggest that microglial dysfunction may be secondary or modulatory. However, important technical and temporal differences between models complicate direct comparisons. Duffy et al.’s model relies on post-weaning Cre activation, bypassing embryonic and early postnatal stages when microglia play key roles in synaptic pruning, vascular development, and immune surveillance [52] (Figure 2). In contrast, the *Cx3cr1^Cre^* driver enables embryonic recombination, allowing assessment of how microglial *Gba1* loss affects brain development and long-term homeostasis [4]. While *Cx3cr1^Cre^* lacks strict microglial specificity leading to recombination in other myeloid lineages, it captures critical developmental windows that are inaccessible to TMEM119-based strategies. Thus, both models offer complementary insights: postnatal neuron-specific deletion establishes causality for neurodegeneration, while embryonic microglial deletion reveals how early microglial programming, substrate buildup, and neuron–glia crosstalk contribute to chronic inflammation. Together, these studies underscore that while neuronal GCase deficiency is sufficient to drive pathology, microglia are not passive bystanders but act as amplifiers of disease, especially with aging and sustained lipid burden. Refining Cre-driver strategies to achieve both developmental access and cell-type specificity remains a critical need. Comparative models targeting *Gba1* deletion prenatally versus postnatally, combined with transcriptomic profiling, will be essential for defining the evolving role of microglia across disease stages and for developing more effective therapeutic platforms for nGD and related lysosomal disorders (Table 2).

Collectively, these studies converge on a unified, hierarchical model for nGD. The central trigger is cell-autonomous GCase loss in neurons, which instigates a cascade of lipid mishandling and cytotoxic stress. While insufficient to initiate disease alone, microglial dysfunction serves as a potent disease amplifier, propagating inflammation and accelerating decline, with its impact contingent on developmental context. This paradigm dictates a two-pronged therapeutic strategy: first, direct correction of the neuronal deficit is paramount to halt disease at its source; second, complementary modulation of microglial responses is necessary to dampen the inflammatory cascade and modify progression. Validating the optimal combination and timing of these approaches will require next-generation models that precisely delineate temporal and cell-specific contributions. Therefore, achieving maximal therapeutic benefit will necessitate interventions that directly restore neuronal health, augmented by parallel efforts to modulate microglial activation and substrate clearance. Defining the precise interplay of these cell types through temporal modeling will be key to unlocking effective, durable treatments for neuronopathic GD. 

## 12. Technical Safeguards and Best Practices—To Avoid Misinterpretation and to Maximize Translational Value, We Recommend That Investigators Using Lineage-Restricted Cre Systems Adopt the Following Standards

1. Reporters and recombination validation: Always include Cre-reporter crosses (e.g., Ai9/tdTomato), genomic PCR, and quantitative GCase activity assays across multiple tissues (brain regions, spleen, liver, muscle, blood myeloid compartments) to document where and when recombination occurs.
2. Temporal controls: If using inducible CreER drivers, carefully report tamoxifen dosing, age at induction, and repopulation dynamics. Where possible, compare embryonic (constitutive) and postnatal (inducible) strategies to separate developmental from maintenance roles.
3. Peripheral checks: Because many “microglial” drivers are active in peripheral myeloid cells at some developmental stages (or after blood–brain barrier disruption), explicitly test for peripheral recombination and consider bone-marrow chimeras or parabiosis experiments to isolate CNS-intrinsic effects.
4. Multiple drivers and rescue approaches: Use at least two independent drivers (e.g., Cx3cr1 and Tmem119, or Cx3cr1 and Sall1, where appropriate) or combine deletion with cell-type rescue experiments to strengthen causal inference.
5. Single-cell and spatial molecular profiling: Integrate single-cell RNA-seq, spatial transcriptomics, and lipidomics to map cell-type–specific substrate accumulation and inflammatory responses; these approaches can reveal whether microglial phenotypes are primary or reactive.
6. Functional readouts across the lifespan: Because microglial contributions may be age-dependent, include long-term behavioral, biomarker (e.g., NfL), and histopathological endpoints.

Implications for therapy. The collective data supports a hierarchical model in which neuronal GCase loss initiates lipid mishandling and cytotoxic stress, and microglial dysfunction amplifies and propagates inflammation. Therapeutically, this suggests that neuron-directed correction should be prioritized in nGD, with adjunctive strategies to enhance microglial substrate clearance and mitigate maladaptive inflammation, as well as to define the optimal timing (developmental vs. adult) of such interventions.

## 13. Chronic Neuronopathic Gaucher Disease Mouse Models

A major gap in the field has been the absence of models that recapitulate progressive, chronic neuronopathic form (GD3) [31]. To address this gap, recent efforts have focused on generating chronic nGD models that better mirror the slow and progressive clinical trajectory of type 3 GD. One such model, *Gba1*^−/−^; Gba^tg^, leverages a doxycycline-inducible Tet-On system to control *Gba1* transgene expression [8]. Despite limited GCase expression in the brain and liver, this model exhibits progressive substrate accumulation (4.2-fold GlcCer increase and elevated GlcSph), coordinated motor deficits beginning around 4–6 months of age, and severe neurodegeneration with widespread gliosis by 8 months. Histological studies revealed neuronal loss in thalamic and cerebellar regions and microgliosis in cortex, thalamus, midbrain, and brainstem, accompanied by skeletal deformities such as kyphosis and bone thinning, hallmarks of type 3 GD [8]. However, the lack of robust GCase re-expression limits its therapeutic testing utility, and the early Dox-induced pathology onset does not fully simulate the adult-onset progression seen in chronic GD patients. In contrast, F213I-inGD mice offer a more controlled, adult-onset model by combining a severe Gba1^F213I^ mutation with tamoxifen-inducible Cre-mediated deletion of a floxed *Gba1* allele. This model exhibits a moderate phenotype, characterized by a lifespan of 50–210 days, progressive neuroinflammation, dopaminergic neuronal loss, α-synuclein accumulation, and peripheral signs such as splenomegaly and Gaucher cell infiltration, while avoiding early-onset lethality. It allows researchers to dissect disease mechanisms, evaluate therapeutic windows, and model Parkinson-like features more faithfully than earlier point mutation models with minimal neurological symptoms [7,13,33,63]. Similarly, the PG9V model created by combining *Gba1*^D409V^ homozygosity with progranulin (Grn) deficiency recapitulates chronic GD pathology in the absence of broad lysosomal gene disruption [64]. PG9V mice develop progressive glycosphingolipid accumulation, CNS and peripheral inflammation, gliosis, and hallmark α-synuclein and β-amyloid aggregation. Importantly, the model reveals a critical role for progranulin in GCase trafficking and BMP homeostasis, with inflammatory and lysosomal abnormalities rescued by treatment with the brain-penetrant PGRN-derived peptide ND7. These features make PG9V a clinically relevant and pharmacologically tractable model for studying *Gba1*-associated neurodegeneration. Collectively, these models *Gba1*^−/−^;*Gba*^tg^, F213I-inGD, and PG9V highlight the growing sophistication of murine systems to emulate chronic nGD. While each has limitations, they reflect different disease onset dynamics, pathological features, and therapeutic applications. Future model refinements, such as adult-onset Cre-loxP systems driven by promoters like Nestin-CreERT2 or CamKIIα-CreERT2, may offer even more precise simulation of late-onset pathology, enabling longitudinal studies of disease progression, biomarker development, and treatment efficacy in chronic nGD (Table 2).

## 14. Modeling Complexities and Comorbidities 

The clinical heterogeneity of GD and its well-established association with Parkinson’s disease underscore that pathogenesis extends beyond the primary enzyme deficiency in macrophages. Mouse models have been crucial for exploring this complexity, revealing how genetic modifiers shape systemic disease and how GCase deficiency in the brain creates a permissive environment for comorbid neurodegeneration. The following sections examine insights from models of genetic modification and the GD-PD link, which together illustrate the intricate networks of lipid metabolism and protein homeostasis that govern disease expression.

## 15. Phenotypic Variability and the Role of *GBA2*: Insights from Double Knockout Models

The *GBA1/GBA2* double knockout (DKO) mouse model was developed to directly test the hypothesis that the non-lysosomal glucosylceramidase *GBA2* acts as a genetic modifier in GD. The phenotypic variability observed in GD, even among patients with identical *GBA1* mutations, highlights the role of additional genetic and metabolic modifiers in influencing disease severity. One such modifier is glucosylceramidase 2 (*GBA2*), a non-lysosomal enzyme involved in GlcCer metabolism. To investigate its contribution to GD1, our lab developed a *GBA1/GBA2* double knockout (DKO) mouse model by crossing *Mx1–Cre^+^;Gba1 ^fl/fl^* mice with *Gba2^−/−^* mice [53]. This model enabled analysis of lysosomal and extra-lysosomal GlcCer metabolism and its impact on disease. In healthy cells, GlcCer generated by GCS is hydrolyzed in lysosomes by *GBA1* or converted by acid ceramidase into GlcSph, which can then be metabolized by *GBA2* to sphingosine [65,66]. In *GBA1* deficiency, both GlcCer and GlcSph accumulate within lysosomes and the cytosol [67], and *GBA2* activity may either compensate for *GBA1* loss or exacerbate disease via excess sphingolipid production (Table 2). Notably, *GBA2* deletion rescued several GD1 phenotypes, including hepatosplenomegaly, cytopenia, and osteopenia in *Mx1–Cre^+^;Gba1 ^fl/fl^* mice. Bone metrics such as bone volume fraction (BV/TV), mineral apposition rate (MAR), and bone formation rate (BFR) were restored, although osteoclast numbers remained unchanged [53]. Despite these improvements, Gaucher cells persisted in the spleen, thymus, and marrow of DKO mice, but were absent in *Gba2^−/−^* mice, suggesting that distinct mechanisms regulate lipid-laden macrophage formation versus systemic disease. Lipidomic analyses revealed persistently elevated GlcCer and GlcSph levels, implicating downstream metabolites such as sphingosine in skeletal pathology. Supporting this, sphingosine but not ceramide or sphingosine-1-phosphate (S1P) reduced preosteoblast viability in a dose-dependent manner, consistent with its known pro-apoptotic effects on bone-forming cells [53]. Beyond systemic disease, GBA2 deletion has shown neuroprotective effects in a mouse model of Niemann–Pick type C (NPC), improving neuronal survival, motor performance, and lifespan [68]. These findings suggest broader relevance for *GBA2* targeting in nGD and Parkinson’s disease (PD), both of which involve *GBA1* dysfunction in the CNS. Complementary studies show that *GBA2* deficiency alone increases GlcCer in spleen, liver, and brain, and that DKO mice accumulate even more GlcCer than single knockouts, suggesting a compensatory relationship between *GBA1* and *GBA2* [69]. *GBA1*-deficient fibroblasts also upregulate *GBA2* expression at the mRNA and protein levels. However, a genetic study found mixed significant association between *GBA2* variants and GD severity, implying that functional, rather than genomic, regulation of GBA2 may influence disease progression [70]. Altogether, the *GBA1/GBA2* DKO model reveals that *GBA2* deletion mitigates systemic and skeletal GD1 features, likely by attenuating toxic sphingolipid accumulation. Persistence of Gaucher cells despite clinical improvement underscores the complexity of disease pathogenesis. These findings position *GBA2* and sphingosine metabolism as promising therapeutic targets for GD1 and potentially nGD and PD (Table 2). Emerging pharmacologic work from the Leiden University group has now advanced this concept to the clinic: they have developed a dual inhibitor targeting both glucosylceramide synthase (GCS) and *GBA2*, which is entering early clinical evaluation as a potential next-generation therapy to simultaneously curb substrate synthesis and extralysosomal GlcCer metabolism [71].

## 16. Gaucher Disease and Parkinson’s Disease Mouse Models

Mouse models of *GBA1* deficiency have been indispensable for dissecting the mechanistic link between GD and Parkinson’s disease (PD), revealing how lysosomal dysfunction, glycosphingolipid accumulation, and α-synuclein pathology intersect to drive neurodegeneration [72,73]. Oligodendrocyte-specific *Gba1* deletion (Cnp1-Cre) impairs myelination and induces early neurodegenerative changes, including demyelination, axonal loss, astrogliosis, and cortical α-synuclein accumulation, highlighting the importance of GCase in glial homeostasis [74]. Several knock-in (KI) models harboring GD/PD-associated *GBA1* mutations (e.g., *Gba1^+/L444P^*, *Gba1^+^/^D409V^*, *Gba1^+/N370S^*) mimic the heterozygous “risk-carrier” state, exhibiting 20–40% reductions in GCase activity and modest GlcCer/GlcSph accumulation, with only subtle lysosomal and oxidative stress in the absence of overt neurodegeneration or motor symptoms. However, these models serve as primed backgrounds that unmask PD-like phenotypes when combined with α-synuclein overexpression (A53T, Thy1-hSNCA), α-synuclein seeds (PFFs), or environmental toxins (e.g., MPTP). To probe these mechanisms further, *GBA1* mutant mice have been combined with α-synuclein stressors, such as overexpression (e.g., Thy1-hSNCA ^A53T^), pre-formed fibrils (PFFs), or toxins such as MPTP. These double-mutant studies demonstrate that *GBA1* deficiency dramatically accelerates α-synuclein aggregation, dopaminergic neuron loss, gliosis, and motor/cognitive deficits [56,70]. A key insight is that GCase loss initiates a cascade of events that begins at the synapse. Collaborative work from Sreeganga Chandra’s lab and ours demonstrated that *Gba1*^+/L444P^ mice exhibit early cognitive impairments and presynaptic defects, including downregulation of synaptic vesicle recycling genes and vesicle loss, without the initial formation of Lewy bodies or nigral degeneration. When crossed with SNCA-transgenic mice, these double mutants exhibit exacerbated motor and cognitive deficits, extensive cortical α-syn pathology, and profound synaptic dysfunction [56]. This supports a model where GCase deficiency first disrupts synaptic integrity and lipid regulation, which then precedes and potentiates α-synuclein aggregation and neurodegeneration. The pathological link between lipids and protein aggregation is underscored by the finding that GlcSph is elevated in young mutant brains and nucleates α-synuclein oligomers, while GlcCer accumulates with age alongside pSer129-α-synuclein inclusions, supporting a lipid-driven model of synucleinopathy [70]. Given its amphipathic structure, α-synuclein shares functional features with apolipoproteins, including the ability to bind acidic phospholipids and glycolipids via membrane-induced helices. We speculate that GlcSph may act as a pathological lipid scaffold akin to an apolipoprotein substrate by directly interacting with α-synuclein and promoting its misfolding and templated aggregation. This hypothesis aligns with our previous findings and adds a plausible biophysical mechanism by which glycosphingolipid accumulation could directly initiate synucleinopathy in *GBA1*-linked PD. This sequence is further validated in toxin-based models, where chronic conduritol B epoxide (CBE) exposure confirms that pharmacological GCase inhibition alone can impair dopamine release, synaptic plasticity, and post-synaptic integrity. While *GBA1* mutant mice alone often show only modest α-synuclein increases without overt Parkinsonism, combined models robustly reproduce cardinal PD features, including Lewy-type inclusions, TH^+^ fiber loss, and behavioral deficits [75]. Notably, some phenotypes, such as cognitive decline, can emerge with the *GBA1* mutation alone, echoing the early cognitive symptoms observed in *GBA1*-PD patients. Collectively, these GD–PD models converge on shared pathophysiological mechanisms, i.e., lysosomal failure, GlcCer/GlcSph accumulation, synaptic vesicle trafficking disruption, α-synuclein aggregation, and neuroinflammation, providing critical platforms for testing GCase-targeted therapies, lipid-lowering agents, and synaptic stabilizers, as well as for biomarker discovery. As emphasized by Farfel-Becker et al., selecting the appropriate model matched to the specific hypothesis or disease stage is essential for translational relevance (Table 2) [31].

## 17. Humanized Mouse Model for Gaucher Disease 

**Figure 3:**
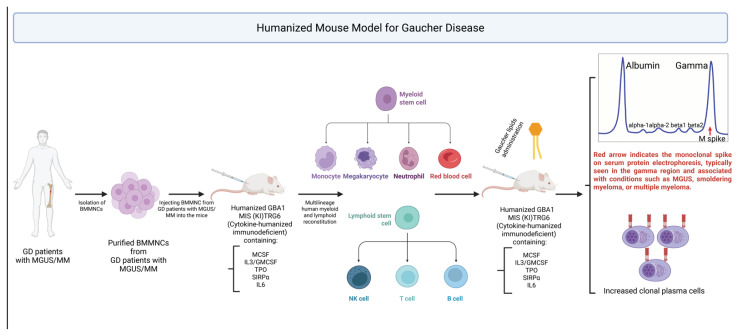
**Humanized Mouse Model for Gaucher Disease (GD)**. To study immune dysregulation in GD, humanized MIS(KI)TRG6 (MISTRG6) mice engineered to express human cytokines (M-CSF, IL-3/GM-CSF, TPO, SIRPα, IL-6), are transplanted with bone marrow mononuclear cells (BM MNCs) from GD patients, including those with MGUS or MM. This enables robust multilineage human immune reconstitution. Upon Gaucher lipid (e.g., GlcSph) administration, mice exhibit a type II NKT cell response, monoclonal IgM spikes, and expansion of CD138⁺ plasma cells. This model recapitulates GD-associated gammopathy and serves as a platform for therapeutic testing.

Humanized Gaucher mouse models now include both “cytokine-humanized” immunodeficient strains and mice bearing human *GBA1* sequences. For example, the MIS(KI)TRG6 (aka MISTRG6) line, in which human M-CSF, IL-3-3/GM-GM-CSF, TPO, and SIRPα (and often IL-6) replace the mouse alleles, supports robust engraftment of human hematopoietic cells. Transplanting bone marrow mononuclear cells (or purified hematopoietic stem cells (HSCs)) from GD patients (including those with GD-associated monoclonal gammopathy of undetermined significance (MGUS)/Multiple Myeloma (MM)) into MIS(KI)TRG6 mice yields multilineage human myeloid and lymphoid reconstitution and permits clonal plasma cell expansion in vivo. In this model, administration of Gaucher lipids (e.g., GlcSph) drives the cognate type II NKT cell response and dramatically boosts the patient-derived monoclonal Ig M-spike and CD138^+^ plasma cell numbers, directly demonstrating antigen-driven gammopathy (Figure 3) [58,59]. These humanized-immune mice also enable testing therapies: for instance, substrate-reduction therapy (eliglustat) reduces the lipid antigen burden and attenuates the clonal Ig spike in preclinical studies [50]. Separately, “humanized-gene” models have been engineered. Jia-Ni Guo et al. generated a knock-in (“mh*Gba1^F^*^213I^”) in which mouse *Gba1* exons 5–7 are replaced by the corresponding human sequence carrying the F213I GD mutation [13]. Heterozygous F213I mice were phenotypically normal, but *Gba1* ^F213I/F213I^ pups showed severely decreased GCase activity and died within 24 h (with abnormal skin). Homozygous lethality mirrors classic knockouts. Prior to this, other mouse models carrying human-relevant *GBA1* alleles had been reported – for example, transgenic or knockin lines bearing common GD mutations such as N370S, L444P or D409V. Together, these published “humanized” GD models, both immune-humanized and gene-humanized, provide complementary platforms to recapitulate human GD immunopathology and test novel therapies (Table 2).

## 18. CRISPR/Cas9 in Gaucher Disease: Modeling Pathology and Correcting Mutations 

CRISPR/Cas9 genome editing has revolutionized GD research by enabling the precise modeling of disease pathogenesis, correction of *GBA1* mutations, and the discovery of regulatory and modifier pathways that contribute to phenotypic variability. Early applications focused on generating isogenic *GBA1* knockout cell lines, such as THP-1 monocytes and U87 glial cells, which recapitulated hallmark GD features, including loss of GCase activity, GlcSph accumulation, α-synuclein aggregation, and activation of inflammatory and stress pathways such as UPR and IL-1β secretion [60]. These in vitro systems have become valuable platforms for screening therapeutic compounds that target lysosomal function, autophagy, or inflammation. In vivo, CRISPR technology has been utilized to generate knock-in models, notably the *Gba1*^F213I/213I^ mouse, which carries a mutation prevalent in East Asian populations [13]. This model mimics human disease, characterized by lipid accumulation, enzyme deficiency, and neuroinflammatory features. Complementary CRISPR-based models in Drosophila and zebrafish have further enabled exploration of lipid trafficking and *GBA1–GBA2* interactions across developmental stages [76]. CRISPR also holds therapeutic promise: one approach involves inserting a functional *GBA1* transgene into the CCR5 safe harbor locus in HSCs under monocyte/macrophage-specific promoters to ensure expression post-transplantation [61]; another corrects mutations such as L444P in patient-derived iPSCs, restoring GCase activity in differentiated macrophages and reducing lipid burden [62]. In vivo delivery using lipid nanoparticles (LNPs) has shown partial restoration of enzyme activity in hepatocytes and hematopoietic cells, indicating potential for non-invasive gene therapy. Beyond modeling and correction, CRISPR has been instrumental in uncovering key disease mechanisms, including impaired autophagy, α-synuclein aggregation, and exaggerated inflammatory signaling in dopaminergic neurons and monocytes, which link GD to Parkinson’s disease. Beyond gene correction, CRISPR screening platforms are now uncovering novel epigenetic and proteostatic modifiers of disease severity, opening entirely new therapeutic avenues. Variability in GD severity has been linked to altered histone acetylation and DNA methylation patterns that affect lysosomal biogenesis, protein folding, and ER stress responses. Yang et al. demonstrated that histone deacetylase inhibitors (HDACis), such as SAHA, enhance the acetylation of Hsp90β, thereby reducing ER retention of mutant GCase and improving lysosomal trafficking [77]. CRISPR-based loss-of-function screens targeting specific HDACs (e.g., HDAC3, HDAC6) or chaperone/co-chaperone genes (e.g., HSPA5, SYVN1, DNMT1) now enable dissection of these regulatory circuits. Moreover, CRISPR interference (CRISPRi) and activation (CRISPRa) approaches enable the fine-tuning of gene expression across proteostasis and epigenetic networks in a tissue-specific manner. Collectively, CRISPR/Cas9 platforms are accelerating our understanding of GD biology by enabling the generation of precise models, the correction of pathogenic variants, and the identification of novel therapeutic targets beyond *GBA1* itself, ushering in a new era of functional genomics and personalized intervention strategies for GD (Table 2).

### AAV-Mediated Gene Therapy for Gaucher Disease

Gene therapy has emerged as a compelling strategy to overcome the fundamental biochemical defect in Gaucher disease (GD) by restoring functional glucocerebrosidase (GCase) activity in affected tissues. Recent work has leveraged adeno-associated virus (AAV) vectors particularly AAV9 and AAVPHP.b to deliver Gba broadly to peripheral organs or selectively to the central nervous system (CNS), addressing the distinct therapeutic challenges of visceral and neuronopathic GD.

AAV9 has been widely utilized due to its favorable biodistribution and its capacity to traverse the blood–brain barrier. Du et al. employed an AAV9 vector encoding Gba under a CMV promoter for systemic expression and a SYN promoter for neuron-specific delivery. In both inducible whole-body and neuron-specific Gba knockout mice, AAV9-mediated gene transfer restored GCase activity, reduced substrate accumulation, attenuated Gaucher cell infiltration, improved neuropathology, and prolonged survival without detectable toxicity [63]. These findings highlight that both systemic and neuron-targeted expression can produce meaningful biochemical and histologic rescue depending on disease context.

Other vector systems helped establish the feasibility of gene therapy in GD. In early proof-of-concept work, Marshall et al. used adenoviral vectors in a chemically induced GD model, demonstrating that even transient GBA expression was sufficient to lower lipid storage and partially normalize cellular function [26]. Although limited by short-term expression and vector immunogenicity, this study laid essential groundwork for subsequent AAV-based approaches. Enquist et al. later used lentiviral vectors to achieve sustained CNS expression of GCase in a neuron-specific GD model, resulting in marked amelioration of neurodegeneration and significant survival benefit [7].

More recently, next-generation neurotropic AAV capsids such as AAVPHP.b have been applied in related lysosomal disorders and are being actively explored for GD. AAVPHP.b exhibits dramatically enhanced CNS penetrance in mice, enabling near pan-neuronal and pan-glial transduction after systemic administration [78]. Combined with cell type–specific promoterssuch as SYN for neurons or GFAP for astrocytes PHP.b vectors offer a powerful platform for dissecting cell-autonomous and non–cell-autonomous mechanisms in GD and for enabling broad CNS correction in neuronopathic subtypes.

As with all gene therapy rescue studies, appropriate interpretation depends on the fidelity of the underlying Cre-loxP models. It is essential to demonstrate—using organ-specific PCR, recombination mapping, reporter alleles, and GCase activity assays—that Gba deletion is complete and accurately restricted to the intended tissues. These safeguards are especially critical when attributing phenotypic correction to the therapeutic vector rather than to incomplete or mosaic recombination, and we discuss these technical considerations in detail later in the review.

Collectively, these studies underscore the therapeutic promise of AAV-mediated gene therapy for both visceral and neuronopathic GD. AAV9’s capacity for dual CNS and systemic targeting, the enhanced neurotropism of AAVPHP.b, and advances in promoter engineering provide unprecedented flexibility in tailoring expression to the pathological domain of interest. Continued improvements in vector design, dosing strategies, and safety profiling are steadily advancing the field toward clinical translation, particularly for neuronopathic forms of GD where current therapies remain insufficient.

## 19. Challenges and Future Directions: Toward Predictive and Patient-Centric Models of Gaucher Disease

The fidelity and translational value of Gaucher disease (GD) mouse models stand at an inflection point. While these systems have yielded foundational insights, their limitations rooted in genetic background, species-specific biology, and environmental variability must now be confronted head-on. The path forward requires not just incremental refinement but a bold reengineering of preclinical platforms that align with the molecular and clinical complexity of human GD.

## 20. Genetic Background and Modifier Networks

Mouse strain background exerts profound effects on GD phenotypes, influencing substrate accumulation, immune activation, neurodegeneration, and therapeutic responsiveness. In nGD models using CBE, early motor decline and shortened survival in C57BL/6JOlaHsd and C3H/HeJ contrast sharply with more resilient 129S1/SvImJ mice. These differences reflect the action of modifier genes such as Grin2b, which modulates excitotoxicity and can be therapeutically targeted with memantine [28]. Copy number variations, such as increased Gbp2b in 129SVE mice, exacerbate macrophage-driven inflammation [79], while immunomodulatory NKT10 cells in C57BL/6 mice buffer against lipid-induced cytokine responses via IL-10 secretion [80]. Our recent work using the Vav-Cre Gba1fl/fl model further underscored the impact of genetic background, revealing strain-dependent variability in Gaucher cell infiltration, immune cell composition, and complement activation signatures [51]. These findings highlight how background genetics can reshape both the hematopoietic landscape and tissue-specific lipid burden in GD. Recognizing and mapping these modifier networks is essential not only for interpreting experimental results but also for anticipating variability in therapeutic responses among patients.

## 21. The Microbiome: A Dynamic Modifier of CNS and Systemic Phenotypes

Beyond host genetics, the microbiome has emerged as a powerful determinant of GD pathophysiology. In a *Drosophila Gba1b^−/−^* model, gut dysbiosis triggered innate immune pathways (Toll, IMD/NF-κB, JAK/STAT), disrupted autophagy, and drove neuroinflammation and glial reactivity [81]. These phenotypes were reversed by germ-free rearing or autophagy induction, demonstrating that the gut–brain–immune axis is not just a modifier but a potential therapeutic target. Given the microbiome’s sensitivity to housing conditions, diet, and even shipping logistics, future studies must rigorously standardize or report microbiome status—or better yet, integrate it as a tractable variable in experimental design.

## 22. Bridging the Genomic Divide: Toward Humanized Mouse Models

A fundamental limitation of murine models is the absence of the human *GBA1* pseudogene (*GBAP1*). In humans, GBAP1 is not only a mutational substrate via gene conversion but also produces protein-coding transcripts that constitute over half of the reads previously assigned to *GBA1*, with tissue- and cell-type–specific expression patterns relevant to lysosomal and neuroimmune regulation [82]. Its complete absence in mice precludes modeling these critical features of GD genomic architecture. Combined with species differences in lipid metabolism, immune cell composition, and vascular calcification pathways, this limits the interpretability of key phenotypes—such as the failure of murine *D409H* models to recapitulate the cardiac manifestations of human GD3c. The future demands humanized *GBA1-GBAP1* locus models, carrying intact regulatory elements and splicing architecture, to allow study of gene conversion, pseudogene-derived transcripts, and regulatory SNPs in an endogenous chromatin context. Only then can we bridge the genotype–phenotype gap between model systems and human patients meaningfully.

## 23. A Call to Action: Defining the Next Generation of GD Models

We call for a coordinated shift toward next-generation murine platforms that are:

- Humanized at the genomic level, incorporating the *GBA1–GBAP1* locus.
- Genetically multiplexed, enabling the modeling of common and rare modifiers through CRISPR-based allelic series.
- Multi-omics-integrated, to capture the dynamic interplay between transcriptomic, lipidomic, and proteomic changes across tissues and developmental stages.
- Microbiome-aware, either through germ-free derivation, co-housing strategies, or defined microbial consortia.
- Mechanistically anchored, with lineage-specific, time-resolved Cre drivers and robust validation protocols.

Such platforms will not only refine our understanding of GD pathogenesis but transform our ability to predict therapeutic response, stratify patients, and uncover vulnerabilities exploitable by gene therapies, substrate reduction agents, and immunomodulators.

Critically, the translational impact of modifier pathways is no longer theoretical. Building on mechanistic insights from *GBA1/GBA2* double knockout mice, recent work from the Leiden group has developed a dual inhibitor of glucosylceramide synthase (GCS) and GBA2, now entering early-stage clinical testing. This convergence of basic discovery and therapeutic translation marks a watershed moment.

In sum, the next era of Gaucher disease research will be defined not by what models can approximate, but by what they can predict. With bold design, rigorous execution, and clinical intent, GD mouse models can evolve from phenomenological surrogates into truly translational engines illuminating biology, guiding intervention, and transforming patient care across all Gaucher subtypes.

## Figures and Tables

**Table 1:** Summary of Gaucher Disease Animal Models.

Model	Strategy	Phenotype	Key Applications & Utility	Limitations	Strain Background	References
Chemically Induced (CBE)	CBE is an irreversible β-glucosidase inhibitor Administered systemically to induce Gaucher pathology	Reduces GCase activity by >90% Induces GlcCer accumulation in brain, liver, spleen Models both acute and chronic GD features Glial activation observed Strain-dependent survival differences Does not induce unfolded protein response (UPR)	Rapid, dose-controllable induction Enables temporal and pharmacological studies latform for neuroinflammation and biomarker (e.g., GPNMB) validation	Lacks UPR induction PK/PD variability Diverges from genetic GD in enzyme trafficking	C57BL/6J and other inbred strains used to study strain-specific effects	[25,26,27,28]
*Gba1*^lnl/lnl^ (Conventional Knockout)	Neo cassette inserted into exons 9 and 10 of *Gba1* gene Results in complete loss of GCase function	NComplete GCase deficiency Severe GlcCer accumulation in liver, brain, lungs, and spleen Lipid-laden macrophages Collodion baby phenotype Neonatal lethality due to defective skin barrier	First genetic Gaucher disease model Demonstrated systemic GCase role Modeled acute type 2 GD with skin and CNS involvement Informed early developmental pathogenesis	Perinatal lethality precludes long-term studies No conditional (tissue-specific) control Skin phenotype dominates over other features	Developed by NIH (1992), typically on C57BL/6 background	[5,29,30]
*Gba1*^lnl/lnl^; K14-Cre (Epidermal-Rescued Conditional KO)	LoxP-flanked *Gba1*lnl allele with Neo insertion Crossed with K14-Cre to restore GCase in skin Enables systemic GCase deficiency while rescuing epidermis	GlcCer accumulation in multiple tissues Lipid-laden neurons Neuroinflammation: activated astrocytes and microglia Partial neonatal survival (~10 days) No prenatal brain defects	Mimics neuropathology of GD type 2 Enables study of acute neurodegeneration and therapeutic window Used in CNS-targeted therapy and BBB penetration studies Supports biomarker development (e.g., ApoE, NfL)	Short lifespan restricts chronic disease modeling Leaky Cre expression outside skin complicates interpretation Limited relevance to prenatal disease stages	Primarily C57BL/6 background	[4,7,31,32]
*Gba1*^Mut/Mut^ Point Knock-In Models (e.g., N370S, D409V, etc.)	CRISPR/homologous recombination Human GBA1 mutation insertion	Mutation-specific GCase activity Early lethality in N370S, RecNciI GlcCer storage in D409H, D409V No cardiac phenotype in D409H	Genotype-phenotype correlation Mutation-specific drug testing Modifier gene studies	Species differences (e.g., N370S lethality) Lacks full human phenotype	NIH-developed, C57BL/6, 129Sv	[33,34,35]
*Gba1*^flox/flox^; Nestin-Cre (Neuronal & Macroglial KO)	Cre-loxP system driven by Nestin promoter Gba1 deleted in neurons and macroglia Microglia retain GCase expression	Delayed-onset neurodegeneration (~2–3 weeks) Seizures and limb rigidity Moderate GlcCer accumulation Absence of lipid-laden microglia GCase activity preserved in microglia	Dissects neuron vs. glial contribution to pathology Enables study of cell-autonomous neurodegeneration Validates microglial protection Models intermediate disease severity	Incomplete CNS GCase deletion Does not fully capture neuron–microglia interactions Still represents early-onset phenotype	C57BL/6	[7]
*Gba1*^flox/flox^; Mx1-Cre (Hematopoietic Conditional KO)	Cre-loxP system with Mx1-Cre, inducible via polyinosinic:polycytidylic acid (poly I:C) Postnatal Gba1 deletion in hematopoietic and mesenchymal lineages The Mistry model uses Gba1^flox/flox; Mx1-Cre mice to conditionally delete exons 8–11 postnatally, achieving > 95% GCase depletion in hematopoietic and mesenchymal stem cells. The Karlsson model employs Gba1^flox/flox; Mx1-Cre mice to delete exons 9–11 postnatally, primarily targeting the hematopoietic lineage.	GCase deficiency in hematopoietic cells Gaucher cells in spleen, liver, bone marrow Splenomegaly and visceral GlcCer/GlcSph accumulation Osteopenia from impaired osteoblastogenesis Disrupted T cell and dendritic cell development No CNS phenotype Biomarkers gpNMB and GlcSph elevated	Recapitulates hematologic and skeletal GD features Avoids perinatal lethality, allowing postnatal studies Supports biomarker discovery and validation (e.g., gpNMB, GlcSph) Critical for studying bone and immune system pathology Used in preclinical lentiviral gene therapy studies	No neurological involvement Variable recombination efficiency across tissues Poly I:C induction may cause off-target effects	C57BL/6 (Karlsson, Mistry)	[2,6,36]
*Gba1*^lnl/lnl^; K14-Cre/Nestin-Cre and Gba1 ^lnl/lnl^; K14-Cre/Cx3cr1-Cre (Conditional Rescue Models—Neuronal & Microglial)	Gba1lnl/lnl mice with K14-Cre (epidermal rescue) Crossed to Nestin-Cre (neuronal) or Cx3cr1-Cre (microglial) drivers Enables selective restoration of GCase in neurons or microglia	>90% reduction of GlcCer and GlcSph in CNS Neuronal rescue (Nestin-Cre): restores microglial homeostasis Microglial rescue (Cx3cr1-Cre): reduces lipid load, monocyte infiltration Distinct effects on neuroinflammation and peripheral immune responses GD3-like behavior persists Motor deficits not fully corrected	Dissects cell-type-specific roles of Gba1 in nGD Highlights neurons as primary GlcCer source Models interdependent neuron–microglia pathology Useful for evaluating cell-targeted gene therapies Demonstrates limits of partial CNS rescue	Partial rescue: does not restore full CNS function Microglial GCase deficiency persists in Nestin-Cre model Residual motor and behavioral deficits	C57BL/6	[4]

**Table 2:** Summary of Gaucher Disease Animal Models.

Model	Strategy	Phenotype	Key Applications & Utility	Limitations	Strain Background	References
*Gba1*^flox/flox^; Cx3cr1-Cre (Constitutive Microglial KO)	Cre-loxP system with Cx3cr1-Cre driver Constitutive recombination in microglia starting during embryogenesis Also affects peripheral monocytes and macrophages due to broad CX3CR1 expression	GCase deletion in microglia Lipid accumulation in CNS Motor deficits observed Recombination may extend to neurons and peripheral immune cells	Models embryonic Gba1 deletion in microglia Enables study of prenatal microglial involvement in GD pathogenesis Useful for examining early neuroimmune activation and systemic immune cell effects	Poor specificity: recombination in non-microglial lineages Cannot isolate microglia-intrinsic contributions Interpretation confounded by peripheral immune involvement	C57BL/6 and other strains used to assess background effects	[4]
*Gba1*^flox/flox^; TMEM119-CreERT2 (Inducible Microglia-Specific KO)	Tamoxifen-inducible Cre-loxP recombination Driven by TMEM119 promoter Enables selective deletion of *Gba1* in mature microglia only	GCase deficiency in postnatally induced, mature brain-resident microglia Preserves GCase activity in neurons, astrocytes, oligodendrocytes, and peripheral immune cells No developmental recombination	High cell-type specificity enables clean mechanistic studies Ideal for transcriptomic profiling of microglial GCase deficiency Useful for modeling adult-onset neuroinflammation Allows separation of adult vs. developmental pathology	Cannot target embryonic or early postnatal microglia May miss critical developmental roles of GCase Requires tamoxifen induction protocol	C57BL/6J and other CreERT2-compatible strains	[4,52]
*Gba1*^flox/flox^; Vav1-Cre (Hematopoietic-Specific KO)	Cre-loxP system using Vav1-Cre to delete *Gba1* in hematopoietic stem cells and progenitors Constitutive GCase loss in all hematopoietic lineages	Bone marrow Gaucher cells Splenomegaly and systemic inflammation GlcCer and GlcSph accumulation Severe thymic/splenic infiltration (notably in VavCre-129 background) Hematopoietic/immune dysregulation: - ↓ MEPs, CD8+ Tmemory, MZ B cells - ↑ Lin−cKit+ progenitors, M2 macrophages RNA-seq: upregulation of GPNMB, lipid metabolism, and immune genes	Faithfully models bone marrow and immune pathology Avoids perinatal lethality Useful for modifier gene discovery Captures lipid-induced immune dysregulation Supports biomarker validation (e.g., GPNMB)	Minimal CNS involvement Weak liver pathology Strong strain-dependent phenotypic differences (e.g., 129 vs. B6)	129X1/SvJ and C57BL/6J (notably different phenotypes)	[51]
*Gba1*^−/−^; Gbatg (Inducible Tet-On/Tet-Off Transgenic Model)	Doxycycline-regulated (Tet-On or Tet-Off) transgene system Enables temporal control of Gba expression *Gba1*^−/−^ background with conditional Gba transgene	Progressive neurological dysfunction: - Ataxia, seizures, motor deficits Skeletal abnormalities: - Kyphosis, brachycephaly Moderate GlcCer and GlcSph accumulation in brain Gliosis and neuronal loss in thalamus and cerebellum	Models type 3 Gaucher disease with CNS progression Enables late-onset and region-specific neurodegeneration studies Useful for timing-specific therapeutic intervention testing Allows modeling of skeletal pathology in GD	Incomplete CNS transgene expression Low GCase levels detectable via WB/RT-PCR Lifespan limited to ~7–10 months Pathology is region-specific, not global	Mixed genetic background (doxycycline-inducible)	[8]
*Gba1*^flox/flox^; Mx1-Cre^+^; Gba2^−/−^ (Double Knockout Model)	Hematopoietic-specific *Gba1* deletion via Mx1–Cre On a *Gba2* ^−/−^ background Designed to assess modifier gene effects and extralysosomal GCase activity	Partial rescue of GD1-like symptoms: - Reduced hepatosplenomegaly, cytopenia, and bone loss Persistent Gaucher cells in spleen and marrow Improved bone architecture: - ↑ BV/TV, MAR, BFR No change in osteoclast numbers Elevated GlcCer and GlcSph Reduced sphingosine toxicity	Explores compensatory or pathogenic roles of GBA2 Separates systemic vs. cellular pathology mechanisms Links sphingolipid metabolism to bone and immune dysfunction Potential model for studying GD1 variability and Parkinson’s-related pathways	Gaucher cells persist despite Gba2 loss No complete lipid clearance CNS effects and rescue mechanisms remain unclear *GBA1* and *GBA2* proximity complicates genetic manipulation	C57BL/6J; poly(I:C)-induced Mx1–Cre; strain-dependent effects reported	[53]
*Gba1*L444P; SNCA^+^ (GD–PD Dual Model)	Knock-in of pathogenic *Gba1*L444P mutation Combined with α-synuclein (SNCA) overexpression Designed to model *GBA1*-linked Parkinson’s and dementia with Lewy bodies (PD/DLB)	Early cognitive and motor deficits Reduced GCase activity GlcCer and GlcSph accumulation Lysosomal dysfunction and pSer129–α-syn aggregation Synaptic vesicle endocytosis (SVE) deficits Loss of clathrin-coated vesicles Microglial and astrocytic priming Downregulation of ER stress-related genes	Models synergistic pathology of *Gba1* mutation and α-syn overexpression Captures early lysosomal and synaptic failure Mimics features of *GBA1*-linked PD and DLB Useful for biomarker discovery and mechanistic studies of protein-lipid interactions Enables testing of therapies targeting SVE, lysosomal function, and lipid-driven α-syn aggregation	Incomplete replication of full PD clinical spectrum Modest glial activation GCase expression not restored Primarily represents early-to-mid stage pathology	C57BL/6J; mixed backgrounds depending on SNCA line	[54,55,56,57]
MIS(KI)TRG6 ^+^ BMMC (Humanized GD Mouse Model)	Engraftment of human bone marrow mononuclear cells (BMMCs) or HSCs into MIS(KI)TRG6 immunodeficient mice Mice are genetically modified to express human cytokines, allowing human immune system reconstitution	Functional human T, B, and myeloid cell reconstitution Activation of lipid-reactive NKT cells Chronic immune activation and inflammation Expansion of plasma cells and GD-associated gammopathy Clonal immunoglobulin (Ig) production Responsive to lipid stimuli (e.g., GlcSph) and eliglustat (SRT) Reduction of M-spike following SRT	Models human immune responses in GD context Enables study of lipid antigen–driven gammopathy and plasma cell dyscrasias Supports biomarker discovery and clonal evolution tracking Used to test substrate reduction therapy (SRT) and immune-modulating interventions Offers platform for exploring GD–multiple myeloma (MM) links	Technically complex and resource-intensive Variable engraftment efficiency CNS involvement not modeled Dependent on variability of human donor material	MIS(KI)TRG6 immunodeficient humanized mouse line	[13,50,58,59]
CRISPR/Cas9-Based GD Models and Therapeutics (In Vivo and In Vitro Systems)	Gene editing of *GBA1* mutations (e.g., F213I) using CRISPR/Cas9 Applied in multiple systems: - Knock-in mice - Zebrafish and Drosophila - Human THP-1 and U87 cell lines - iPSCs and hematopoietic stem cells (HSCs) Includes CRISPRa/i for epigenetic modulation	GCase deficiency and GlcSph accumulation ER stress and α-synuclein aggregation Proinflammatory cytokine production (IL-1β, TNFα) Knock-in mice show lipid buildup and neurologic deficits iPSC-derived macrophages regain GCase activity after gene correction In vivo CRISPR therapy partially restores GCase function CRISPRa/i identifies roles for HDACs, Hsp90, chaperones	Precision modeling of patient-specific *GBA*1 mutations Platform for gene correction and therapeutic testing High-throughput drug screening and epigenetic interrogation Useful in Parkinson’s disease and neuroinflammation studies Bridges basic disease modeling and translational gene therapy	Incomplete correction with in vivo delivery Risk of off-target effects Epigenetic tools (CRISPRa/i) require strict calibration Species-specific differences in pathway behavior	Mice (e.g., F213I KI), zebrafish, Drosophila, THP-1, U87, human iPSC-derived cells	[60,61,62]

## Data Availability

No new data were created or analyzed in this study. Data sharing is not applicable to this article.

## References

[1] Grabowski G.A., Petsko G.A., Kolodny E.H., Valle D.L., Antonarakis S., Ballabio A., Beaudet A.L., Mitchell G.A. (2019). Gaucher Disease. The Online Metabolic and Molecular Bases of Inherited Disease.

[2] Mistry P.K., Liu J., Yang M., Nottoli T., McGrath J., Jain D., Zhang K., Keutzer J., Chuang W.-L., Mehal W.Z. (2010). Glucocerebrosidase gene-deficient mouse recapitulates Gaucher disease displaying cellular and molecular dysregulation beyond the macrophage. Proc. Natl. Acad. Sci. USA.

[3] Ginhoux F., Lim S., Hoeffel G., Low D., Huber T. (2013). Origin and differentiation of microglia. Front. Cell. Neurosci..

[4] Boddupalli C.S., Nair S., Belinsky G., Gans J., Teeple E., Nguyen T.-H., Mehta S., Guo L., Kramer M.L., Ruan J. (2022). Neuroinflammation in neuronopathic Gaucher disease: Role of microglia and NK cells, biomarkers, and response to substrate reduction therapy. Elife.

[5] Tybulewicz V.L., Tremblay M.L., LaMarca M.E., Willemsen R., Stubblefield B.K., Winfield S., Zablocka B., Sidransky E., Martin B.M., Huang S.P. (1992). Animal model of Gaucher’s disease from targeted disruption of the mouse glucocerebrosidase gene. Nature.

[6] Enquist I.B., Nilsson E., Ooka A., Mansson J.E., Olsson K., Ehinger M., Brady R.O., Richter J., Karlsson S. (2006). Effective cell and gene therapy in a murine model of Gaucher disease. Proc. Natl. Acad. Sci. USA.

[7] Enquist I.B., Lo Bianco C., Ooka A., Nilsson E., Mansson J.E., Ehinger M., Richter J., Brady R.O., Kirik D., Karlsson S. (2007). Murine models of acute neuronopathic Gaucher disease. Proc. Natl. Acad. Sci. USA.

[8] Pewzner-Jung Y., Joseph T., Blumenreich S., Vardi A., Ferreira N.S., Cho S.M., Eilam R., Tsoory M., Biton I.E., Brumfeld V. (2021). Brain pathology and cerebellar purkinje cell loss in a mouse model of chronic neuronopathic Gaucher disease. Prog. Neurobiol..

[9] Grabowski G.A., Antommaria A.H.M., Kolodny E.H., Mistry P.K. (2021). Gaucher disease: Basic and translational science needs for more complete therapy and management. Mol. Genet. Metab..

[10] Woo E.G., Tayebi N., Sidransky E. (2021). Next-Generation Sequencing Analysis of GBA1: The Challenge of Detecting Complex Recombinant Alleles. Front. Genet..

[11] O’Neill R.R., Tokoro T., Kozak C.A., Brady R.O. (1989). Comparison of the chromosomal localization of murine and human glucocerebrosidase genes and of the deduced amino acid sequences. Proc. Natl. Acad. Sci. USA.

[12] Tayebi N., Stubblefield B.K., Park J.K., Orvisky E., Walker J.M., LaMarca M.E., Sidransky E. (2003). Reciprocal and nonreciprocal recombination at the glucocerebrosidase gene region: Implications for complexity in Gaucher disease. Am. J. Hum. Genet..

[13] Guo J.N., Guan M., Jiang N., Li N., Li Y.J., Zhang J., Ma D. (2022). Establishment and Phenotypic Analysis of the Novel Gaucher Disease Mouse Model With the Partially Humanized Gba1 Gene and F213I Mutation. Front. Genet..

[14] Mistry P.K. (1995). Genotype/phenotype correlations in Gaucher’s disease. Lancet.

[15] Deen M.C., Zhu Y., Gros C., Na N., Gilormini P.A., Shen D.L., Bhosale S., Anastasi N., Wang R., Shan X. (2022). A versatile fluorescence-quenched substrate for quantitative measurement of glucocerebrosidase activity within live cells. Proc. Natl. Acad. Sci. USA.

[16] Abu-Remaileh M., Wyant G.A., Kim C., Laqtom N.N., Abbasi M., Chan S.H., Freinkman E., Sabatini D.M. (2017). Lysosomal metabolomics reveals V-ATPase- and mTOR-dependent regulation of amino acid efflux from lysosomes. Science.

[17] Capecchi M.R. (2022). The origin and evolution of gene targeting. Dev. Biol..

[18] Gould S.E., Junttila M.R., de Sauvage F.J. (2015). Translational value of mouse models in oncology drug development. Nat. Med..

[19] Cabrera-Salazar M., Bercury S., Ziegler R., Marshall J., Hodges B., Chuang W.-L., Pacheco J., Li L., Cheng S., Scheule R. (2010). Intracerebroventricular delivery of glucocerebrosidase reduces substrates and increases lifespan in a mouse model of neuronopathic Gaucher disease. Exp. Neurol..

[20] Chang M., Cooper J.D., Sleat D.E., Cheng S.H., Dodge J.C., Passini M.A., Lobel P., Davidson B.L. (2008). Intraventricular enzyme replacement improves disease phenotypes in a mouse model of late infantile neuronal ceroid lipofuscinosis. Mol. Ther..

[21] Schiffmann R., Cox T.M., Dedieu J.F., Gaemers S.J.M., Hennermann J.B., Ida H., Mengel E., Minini P., Mistry P., Musholt P.B. (2023). Venglustat combined with imiglucerase for neurological disease in adults with Gaucher disease type 3: The LEAP trial. Brain.

[22] Marshall J., Sun Y., Bangari D.S., Budman E., Park H., Nietupski J.B., Allaire A., Cromwell M.A., Wang B., Grabowski G.A. (2016). CNS-accessible Inhibitor of Glucosylceramide Synthase for Substrate Reduction Therapy of Neuronopathic Gaucher Disease. Mol. Ther..

[23] Mistry P.K., Lukina E., Ben Turkia H., Shankar S.P., Baris Feldman H., Ghosn M., Mehta A., Packman S., Lau H., Petakov M. (2021). Clinical outcomes after 4.5 years of eliglustat therapy for Gaucher disease type 1: Phase 3 ENGAGE trial final results. Am. J. Hematol..

[24] Sawkar A.R., Cheng W.-C., Beutler E., Wong C.-H., Balch W.E., Kelly J.W. (2002). Chemical chaperones increase the cellular activity of N370S β-glucosidase: A therapeutic strategy for Gaucher disease. Proc. Natl. Acad. Sci. USA.

[25] Stephens M.C., Bernatsky A., Burachinsky V., Legler G., Kanfer J.N. (1978). The Gaucher mouse: Differential action of conduritol B epoxide and reversibility of its effects. J. Neurochem..

[26] Marshall J., McEachern K.A., Kyros J.A., Nietupski J.B., Budzinski T., Ziegler R.J., Yew N.S., Sullivan J., Scaria A., van Rooijen N. (2002). Demonstration of feasibility of in vivo gene therapy for Gaucher disease using a chemically induced mouse model. Mol. Ther..

[27] Vardi A., Zigdon H., Meshcheriakova A., Klein A.D., Yaacobi C., Eilam R., Kenwood B.M., Rahim A.A., Massaro G., Merrill A.H. (2016). Delineating pathological pathways in a chemically induced mouse model of Gaucher disease. J. Pathol..

[28] Klein A.D., Ferreira N.S., Ben-Dor S., Duan J., Hardy J., Cox T.M., Merril A.H., Futerman A.H. (2016). Identification of Modifier Genes in a Mouse Model of Gaucher Disease. Cell Rep..

[29] Sidransky E., Ginns E.I. (1997). Gaucher’s disease: The best laid schemes of mice and men. Baillieres Clin. Haematol..

[30] Holleran W.M., Ginns E.I., Menon G.K., Grundmann J.U., Fartasch M., McKinney C.E., Elias P.M., Sidransky E. (1994). Consequences of beta-glucocerebrosidase deficiency in epidermis. Ultrastructure and permeability barrier alterations in Gaucher disease. J. Clin. Investig..

[31] Farfel-Becker T., Vitner E.B., Futerman A.H. (2011). Animal models for Gaucher disease research. Dis. Models Mech..

[32] Massaro G., Mattar C.N.Z., Wong A.M.S., Sirka E., Buckley S.M.K., Herbert B.R., Karlsson S., Perocheau D.P., Burke D., Heales S. (2018). Fetal gene therapy for neurodegenerative disease of infants. Nat. Med..

[33] Xu Y.H., Quinn B., Witte D., Grabowski G.A. (2003). Viable mouse models of acid beta-glucosidase deficiency: The defect in Gaucher disease. Am. J. Pathol..

[34] Sun Y., Quinn B., Witte D.P., Grabowski G.A. (2005). Gaucher disease mouse models: Point mutations at the acid beta-glucosidase locus combined with low-level prosaposin expression lead to disease variants. J. Lipid Res..

[35] Liu Y., Suzuki K., Reed J.D., Grinberg A., Westphal H., Hoffmann A., Döring T., Sandhoff K., Proia R.L. (1998). Mice with type 2 and 3 Gaucher disease point mutations generated by a single insertion mutagenesis procedure (SIMP). Proc. Natl. Acad. Sci. USA.

[36] Kramer G., Wegdam W., Donker-Koopman W., Ottenhoff R., Gaspar P., Verhoek M., Nelson J., Gabriel T., Kallemeijn W., Boot R.G. (2016). Elevation of glycoprotein nonmetastatic melanoma protein B in type 1 Gaucher disease patients and mouse models. FEBS Open Bio.

[37] Murugesan V., Liu J., Yang R., Lin H., Lischuk A., Pastores G., Zhang X., Chuang W.-L., Mistry P.K. (2018). Validating glycoprotein non-metastatic melanoma B (gpNMB, osteoactivin), a new biomarker of Gaucher disease. Blood Cells Mol. Dis..

[38] Moloney E.B., Moskites A., Ferrari E.J., Isacson O., Hallett P.J. (2018). The glycoprotein GPNMB is selectively elevated in the substantia nigra of Parkinson’s disease patients and increases after lysosomal stress. Neurobiol. Dis..

[39] Kurzawa-Akanbi M., Hanson P.S., Blain P.G., Lett D.J., McKeith I.G., Chinnery P.F., Morris C.M. (2012). Glucocerebrosidase mutations alter the endoplasmic reticulum and lysosomes in Lewy body disease. J. Neurochem..

[40] Panicker L.M., Srikanth M.P., Castro-Gomes T., Miller D., Andrews N.W., Feldman R.A. (2018). Gaucher disease iPSC-derived osteoblasts have developmental and lysosomal defects that impair bone matrix deposition. Hum. Mol. Genet..

[41] Mistry P.K., Weinreb N.J., Kaplan P., Cole J.A., Gwosdow A.R., Hangartner T. (2011). Osteopenia in Gaucher disease develops early in life: Response to imiglucerase enzyme therapy in children, adolescents and adults. Blood Cells Mol. Dis..

[42] Liu J., Halene S., Yang M., Iqbal J., Yang R., Mehal W.Z., Chuang W.-L., Jain D., Yuen T., Sun L. (2012). Gaucher disease gene GBA functions in immune regulation. Proc. Natl. Acad. Sci. USA.

[43] Ch’en I.Y., Lynch D.A., Shroyer K.R., Schwarz M.I. (1993). Gaucher’s disease. An unusual cause of intrathoracic extramedullary hematopoiesis. Chest.

[44] Stein P., Malhotra A., Haims A., Pastores G.M., Mistry P.K. (2010). Focal splenic lesions in type I Gaucher disease are associated with poor platelet and splenic response to macrophage-targeted enzyme replacement therapy. J. Inherit. Metab. Dis..

[45] Mistry P.K., Taddei T., vom Dahl S., Rosenbloom B.E. (2013). Gaucher disease and malignancy: A model for cancer pathogenesis in an inborn error of metabolism. Crit. Rev. Oncog..

[46] Rosenbloom B.E., Cappellini M.D., Weinreb N.J., Dragosky M., Revel-Vilk S., Batista J.L., Sekulic D., Mistry P.K. (2022). Cancer risk and gammopathies in 2123 adults with Gaucher disease type 1 in the International Gaucher Group Gaucher Registry. Am. J. Hematol..

[47] Rosenbloom B.E., Weinreb N.J., Zimran A., Kacena K.A., Charrow J., Ward E. (2005). Gaucher disease and cancer incidence: A study from the Gaucher Registry. Blood.

[48] Pavlova E.V., Wang S.Z., Archer J., Dekker N., Aerts J.M., Karlsson S., Cox T. (2013). B cell lymphoma and myeloma in murine Gaucher’s disease. J. Pathol..

[49] Nair S., Boddupalli C.S., Verma R., Liu J., Yang R., Pastores G.M., Mistry P.K., Dhodapkar M.V. (2015). Type II NKT-TFH cells against Gaucher lipids regulate B-cell immunity and inflammation. Blood.

[50] Nair S., Branagan A.R., Liu J., Boddupalli C.S., Mistry P.K., Dhodapkar M.V. (2016). Clonal Immunoglobulin against Lysolipids in the Origin of Myeloma. N. Engl. J. Med..

[51] Belinsky G., Ruan J., Fattahi N., Mehta S., Boddupalli C.S., Mistry P.K., Nair S. (2025). Modeling Bone Marrow Microenvironment and Hematopoietic Dysregulation in Gaucher Disease through Vav-Cre Mediated GBA1 Deletion. Hum. Mol. Genet..

[52] Duffy H.B.D., Byrnes C., Zhu H., Tuymetova G., Lee Y.T., Platt F.M., Proia R.L. (2024). Deletion of Gba in neurons, but not microglia, causes neurodegeneration in a Gaucher mouse model. JCI Insight.

[53] Mistry P.K., Liu J., Sun L., Chuang W.-L., Yuen T., Yang R., Lu P., Zhang K., Li J., Keutzer J. (2014). Glucocerebrosidase 2 gene deletion rescues type 1 Gaucher disease. Proc. Natl. Acad. Sci. USA.

[54] Mazzulli J.R., Xu Y.H., Sun Y., Knight A.L., McLean P.J., Caldwell G.A., Sidransky E., Grabowski G.A., Krainc D. (2011). Gaucher disease glucocerebrosidase and alpha-synuclein form a bidirectional pathogenic loop in synucleinopathies. Cell.

[55] Sardi S.P., Clarke J., Kinnecom C., Tamsett T.J., Li L., Stanek L.M., Passini M.A., Grabowski G.A., Schlossmacher M.G., Sidman R.L. (2011). CNS expression of glucocerebrosidase corrects alpha-synuclein pathology and memory in a mouse model of Gaucher-related synucleinopathy. Proc. Natl. Acad. Sci. USA.

[56] Taguchi Y.V., Liu J., Ruan J., Pacheco J., Zhang X., Abbasi J., Keutzer J., Mistry P.K., Chandra S.S. (2017). Glucosylsphingosine Promotes alpha-Synuclein Pathology in Mutant GBA-Associated Parkinson’s Disease. J. Neurosci..

[57] Zunke F., Moise A.C., Belur N.R., Gelyana E., Stojkovska I., Dzaferbegovic H., Toker N.J., Jeon S., Fredriksen K., Mazzulli J.R. (2018). Reversible Conformational Conversion of alpha-Synuclein into Toxic Assemblies by Glucosylceramide. Neuron.

[58] Nair S., Bar N., Xu M.L., Dhodapkar M., Mistry P.K. (2020). Glucosylsphingosine but not Saposin C, is the target antigen in Gaucher disease-associated gammopathy. Mol. Genet. Metab..

[59] Nair S., Sng J., Boddupalli C.S., Seckinger A., Chesi M., Fulciniti M., Zhang L., Rauniyar N., Lopez M., Neparidze N. (2018). Antigen-mediated regulation in monoclonal gammopathies and myeloma. JCI Insight.

[60] Pavan E., Ormazabal M., Peruzzo P., Vaena E., Rozenfeld P., Dardis A. (2020). CRISPR/Cas9 Editing for Gaucher Disease Modelling. Int. J. Mol. Sci..

[61] Scharenberg S.G., Poletto E., Lucot K.L., Colella P., Sheikali A., Montine T.J., Porteus M.H., Gomez-Ospina N. (2020). Engineering monocyte/macrophage-specific glucocerebrosidase expression in human hematopoietic stem cells using genome editing. Nat. Commun..

[62] Ramalingam S., Kumar A., Krug S., Mohan H., Rao D.N., Bishai W.R., Chandrasegaran S. (2023). CRISPR Correction of the GBA Mutation in Human-Induced Pluripotent Stem Cells Restores Normal Function to Gaucher Macrophages and Increases Their Susceptibility to Mycobacterium tuberculosis. J. Infect. Dis..

[63] Du S., Ou H., Cui R., Jiang N., Zhang M., Li X., Ma J., Zhang J., Ma D. (2019). Delivery of Glucosylceramidase Beta Gene Using AAV9 Vector Therapy as a Treatment Strategy in Mouse Models of Gaucher Disease. Hum. Gene Ther..

[64] Lin Y., Zhao X., Liou B., Fannin V., Zhang W., Setchell K.D.R., Wang X., Pan D., Grabowski G.A., Liu C.-J. (2024). Intrinsic link between PGRN and Gba1 D409V mutation dosage in potentiating Gaucher disease. Hum. Mol. Genet..

[65] Yildiz Y., Matern H., Thompson B., Allegood J.C., Warren R.L., Ramirez D.M., Hammer R.E., Hamra F.K., Matern S., Russell D.W. (2006). Mutation of beta-glucosidase 2 causes glycolipid storage disease and impaired male fertility. J. Clin. Investig..

[66] Boot R.G., Verhoek M., Donker-Koopman W., Strijland A., van Marle J., Overkleeft H.S., Wennekes T., Aerts J.M.F. (2007). Identification of the non-lysosomal glucosylceramidase as beta-glucosidase 2. J. Biol. Chem..

[67] Hein L.K., Meikle P.J., Hopwood J.J., Fuller M. (2007). Secondary sphingolipid accumulation in a macrophage model of Gaucher disease. Mol. Genet. Metab..

[68] Marques A.R., Aten J., Ottenhoff R., van Roomen C.P., Herrera Moro D., Claessen N., Veloz M.F.V., Zhou K., Lin Z., Mirzaian M. (2015). Reducing GBA2 activity ameliorates neuropathology in Niemann-Pick type C mice. PLoS ONE.

[69] Yildiz Y., Hoffmann P., Vom Dahl S., Breiden B., Sandhoff R., Niederau C., Horwitz M., Karlsson S., Filocamo M., Elstein D. (2013). Functional and genetic characterization of the non-lysosomal glucosylceramidase 2 as a modifier for Gaucher disease. Orphanet J. Rare Dis..

[70] Kim S., Kwon S.H., Kam T.I., Panicker N., Karuppagounder S.S., Lee S., Lee J.H., Kim W.R., Kook M., Foss C.A. (2019). Transneuronal Propagation of Pathologic alpha-Synuclein from the Gut to the Brain Models Parkinson’s Disease. Neuron.

[71] Gehin M., Melchior M., Welford R.W.D., Sidharta P.N., Dingemanse J. (2021). Assessment of Target Engagement in a First-in-Human Trial with Sinbaglustat, an Iminosugar to Treat Lysosomal Storage Disorders. Clin. Transl. Sci..

[72] Shiner T., Mirelman A., Gana Weisz M., Bar-Shira A., Ash E., Cialic R., Nevler N., Gurevich T., Bregman N., Orr-Urtreger A. (2016). High Frequency of GBA Gene Mutations in Dementia With Lewy Bodies Among Ashkenazi Jews. JAMA Neurol..

[73] Sidransky E., Lopez G. (2012). The link between the GBA gene and parkinsonism. Lancet Neurol..

[74] Gregorio I., Russo L., Torretta E., Barbacini P., Contarini G., Pacinelli G., Bizzotto D., Moriggi M., Braghetta P., Papaleo F. (2024). GBA1 inactivation in oligodendrocytes affects myelination and induces neurodegenerative hallmarks and lipid dyshomeostasis in mice. Mol. Neurodegener..

[75] Wallom K.L., Fernandez-Suarez M.E., Priestman D.A., Te Vruchte D., Huebecker M., Hallett P.J., Isacson O., Platt F.M. (2022). Glycosphingolipid metabolism and its role in ageing and Parkinson’s disease. Glycoconj. J..

[76] Wang L., Lin G., Zuo Z., Li Y., Byeon S.K., Pandey A., Bellen H.J. (2022). Neuronal activity induces glucosylceramide that is secreted via exosomes for lysosomal degradation in glia. Sci. Adv..

[77] Yang C., Rahimpour S., Lu J., Pacak K., Ikejiri B., Brady R.O., Zhuang Z. (2013). Histone deacetylase inhibitors increase glucocerebrosidase activity in Gaucher disease by modulation of molecular chaperones. Proc. Natl. Acad. Sci. USA.

[78] Morabito G., Giannelli S.G., Ordazzo G., Bido S., Castoldi V., Indrigo M., Cabassi T., Cattaneo S., Luoni M., Cancellieri C. (2017). AAV-PHP.B-Mediated Global-Scale Expression in the Mouse Nervous System Enables GBA1 Gene Therapy for Wide Protection from Synucleinopathy. Mol. Ther..

[79] Clough B., Finethy R., Khan R.T., Fisch D., Jordan S., Patel H., Coers J., Frickel E.-M. (2019). C57BL/6 and 129 inbred mouse strains differ in Gbp2 and Gbp2b expression in response to inflammatory stimuli in vivo. Wellcome Open Res..

[80] Sag D., Krause P., Hedrick C.C., Kronenberg M., Wingender G. (2014). IL-10-producing NKT10 cells are a distinct regulatory invariant NKT cell subset. J. Clin. Investig..

[81] Atilano M.L., Hull A., Romila C.A., Adams M.L., Wildfire J., Urena E., Dyson M., Ivan-Castillo-Quan J., Partridge L., Kinghorn K.J. (2023). Autophagic dysfunction and gut microbiota dysbiosis cause chronic immune activation in a Drosophila model of Gaucher disease. PLoS Genet..

[82] Gustavsson E.K., Sethi S., Gao Y., Brenton J.W., Garcia-Ruiz S., Zhang D., Garza R., Reynolds R.H., Evans J.R., Chen Z. (2024). The annotation of GBA1 has been concealed by its protein-coding pseudogene GBAP1. Sci. Adv..

